# Bioprocess development for enhanced endoglucanase production by newly isolated bacteria, purification, characterization and in-vitro efficacy as anti-biofilm of *Pseudomonas aeruginosa*

**DOI:** 10.1038/s41598-021-87901-9

**Published:** 2021-05-07

**Authors:** Atef M. Ibrahim, Ragaa A. Hamouda, Noura El-Ahmady El-Naggar, Fatma M. Al-Shakankery

**Affiliations:** 1 Microbial Biotechnology Department, Genetic Engineering and Biotechnology Research Institute, University of Sadat City, Menoufia Governorate, 22857 Egypt; 2 Department of Bioprocess Development, Genetic Engineering and Biotechnology Research Institute, City of Scientific Research and Technological Applications (SRTA-City), New Borg El-Arab City, 21934 Alexandria Egypt

**Keywords:** Biochemistry, Biotechnology, Industrial microbiology

## Abstract

Endoglucanase producing bacteria were isolated from Egyptian soils and the most active bacterial strain was identified as *Bacillus subtilis* strain Fatma/1. Plackett–Burman statistical design was carried out to assess the effect of seven process variables on endoglucanase production. Carboxymethyl cellulose (CMC), yeast extract and peptone were the most significant variables that enhanced the endoglucanase production and thus were selected for further optimization using face-centered central composite design. The highest yield of endoglucanase (32.37 U/mL) was obtained in run no. 9, using 18 g/L CMC, 8 g/L peptone, 7 g/L yeast extract and 0.1 g/L FeSO_4_.7H_2_O. The optimized medium showed about eightfold increase in endoglucanase production compared to the unoptimized medium. The produced crude enzyme was further purified by ammonium sulfate precipitation, then DEAE-Sepharose CL6B column. The purified enzyme was shown to have a molecular weight of 37 kDa. The enzyme showed maximum activity at pH 8.0, temperature of 50 °C, incubation time of 60 min. The half-life time (T_1/2_) was 139.53 min at 50 °C, while being 82.67 min at 60 °C. Endoglucanase at concentration of 12 U/mL effectively removed 84.61% of biofilm matrix of *Pseudomonas aeruginosa* with marked reduction in carbohydrate content of the biofilm from 63.4 to 7.9 μg.

## Introduction

Cellulose is one of the earth's bio compounds that occurs in vast quantities^1,2^. Cellulose is an unbranched polymer of glucose monomers linked by β-1,4- glycosidic bonds with a conformation that is relatively resistant to hydrolysis^3^. The need for novel cellulases that can degrade cellulose material into simple forms, e.g. glucose has been required for harnessing energy from cellulose^3^. While it is easy to break down cellulose into simple sugars using conventional chemical methods, the enzymatic process is considered economical, non-polluting and environmentally-save^3,4^.

Cellulase can degrade cellulose molecule by hydrolyzing β-1–4 glycosidic bonds present in the structure. For the full hydrolysis of cellulose into glucose, three enzymes are involved: endoglucanase (EC 3.2.1.4), exoglucanase (EC 3.2.1.91) and β-glucosidase (EC 3.2.1.21)^5^. Endoglucanases are suggested to initiate a random attack at several inner sites in cellulose compound, making the polymer more exposed to hydrolysis by other cellohydrolases^5^.

Cellulases are produced by a vast array of microorganisms while growing on cellulolytic substrates^6^. Enzymes from microbial origin are preferred over enzymes from animal or plant sources because they are cheaper with well-known enzyme production and secretion systems^7^. Bacterial cellulases are gaining interest due to their biodiversity, easy recovery of the product and their ability to make enzymes that can resist the extreme conditions in the surrounding environment^8,9^. Out of all *Bacillus* species, *Bacillus subtilis* continues to be a significant working house owing to its ability to release tremendous amounts of lytic enzymes and high adaptability to changing environmental conditions^10,11^.

Microbial cellulases have many potential industrial and biotechnological applications, and thus are highly demanded^12,13^. Cellulase has become the world's third group of enzymes utilized in the industrial sector^14^. The rising concerns about producing cellulases is due to their various implementations in textile, bioethanol, detergent, food, feed, leather, pulp and papermaking processes^15–19^. Cellulase serves as a digestive aid that digests fibers and this helps to cure digestive issues such as malabsorption. Since cellulose fiber is poorly digested by humans^20^. It may be used for the treatment of phytobezoars, a type of indigestible cellulose mass trapped in the human gut^21^. Cellulases can efficiently remove biofilms of pathogenic bacteria grown on medical devices^22^. It is also used for bioremediation processes, treatment of waste water and single cell protein as well^23^.

The process of enzyme purification is of great concern in order to gain knowledge about structural and functional properties and to predict their applications. A given enzyme`s ultimate degree of purity depends upon its end use. The aim behind the purification strategy is to obtain the largest possible yield of the desired enzyme with the best catalytic activity^24^. Some studies involved the direct application of fungal or bacterial cellulases, mainly from *Bacillus* species, as antibiofilm agents for medical implants^25^, diverse prostethic materials^22^, treatment of nosocomial infections^26^.

An open wound is a suitable niche for colonizing microbes^27^. Elgharably et al.^28^ stated that biofilm development, by a distinct pathogen is a key element involved in the colonization and persistence of its infections, as it protects the bacteria from the host immune response and provides a protective barrier that enables antimicrobial resistance^29^. Alternatives to antibiotics, such as methods for biofilm degradation, are seriously needed to combat major pathogenic bacteria^30^. A novel strategy for degradation of biofilms is required for effective microbial control.

In the current study, a novel strain of *Bacillus subtilis* Fatma/1 was isolated from soil and screened for the synthesis of endoglucanase. Enzyme production was improved by optimizing fermentation conditions. The optimization of production processess was performed using a multistep experimental scheme. Initially the physical parameters were optimized. Later, a two-level multifactorial Plackett–Burman statistical design was applied to determine appropriate medium constituents which were then applied for further optimization using face centered central composite design. The produced enzyme was purified using ammonium sulphate precipitation and ion-exchange chromatography via DEAE-Sepharose CL6B column and tested for its inhibiting effect against biofilm of two nosocomial pathogens.

## Materials and methods

### Soil samples collection, culture medium and isolation of bacteria

Soil samples were collected in sterile containers from the area of Wadi El Natrun, Beheira Governorate, Egypt, and kept at 4 °C before bacterial isolation. The place is located between longitudes, 30°02′ and 30°29′ E and latitudes, 30°16′ and 30°32′ N. The samples containing cellulolytic organisms was cultured on carboxymethyl cellulose agar medium^31^ composed of: (g/L): Carboxymethyl cellulose (CMC), 10; KH_2_PO_4_, 4; Na_2_HPO_4_, 4; tryptone, 2; MgSO_4_·7H_2_O, 0.2; CaCl_2_·2H_2_O, 0.001; FeSO_4_·7H_2_O, 0.004; Agar, 15 at neutral pH. Ten-fold serial dilution of each soil sample was prepared using sterile distilled water as diluent. Aliquots (0.1 mL) of diluted samples were plated out using the pour plate method. The aliquots were cultured in duplicates and the inoculated plates were incubated for 72 h at a temperature of 37 °C. Different colonies were picked and sub-cultured on nutrient agar to obtain pure cultures.

### Screening of endoglucanase producing microorganisms

Isolates were grown on Luria Bertani (LB) medium at 37 °C for 24 h. The isolates were then replica streaked on plates of agar media containing carboxymethyl cellulose (CMC), 10; KH_2_PO_4_, 4; Na_2_HPO_4_, 4; tryptone, 2; MgSO_4_·7H_2_O, 0.2; CaCl_2_·2H_2_O, 0.001; FeSO_4_·7H_2_O, 0.004; NaCl, 5; Agar, 15 g/L. pH was adjusted to 7.0. Plates were incubated at 37 °C for 48 h.

Zones of hydrolysis are made clear by flooding the agar plates containing carboxymethyl cellulose with a solution of 0.1% Congo red for 15 min. The plates were then washed using a solution of 1 M NaCl. Diameters of clear zones around colonies were measured. This was used for screening endoglucanase-producing bacteria. The ratio of the diameter of the clear zone and the colony (cellulolytic index) of different isolates was determined via the method of Ferbiyanto et al.^32^. The following equation was applied:

1 $$ {\text{Cellulolytic index = }}\frac{{\text{Diameter of clear zone - Diameter of bacterial colony}}}{{\text{Diameter of bacterial colony}}} \, $$

Isolate with the highest cellulolytic index was selected for further studies.

### Identification of the bacterial isolate

#### Morphological and biochemical characterization

Different morphological characteristics, microscopic examination and biochemical tests such as Gram stain, catalase production, starch hydrolysis, growth on citrate, nitrate reduction and sugar fermentation were performed for identification^33^.

#### Electron microscopy

The bacterial strain Fatma/1 was dehydrated, coated with gold and examined at different magnifications with Analytical Scanning Electron Microscope Jeol JSM-6510 lv operating at 20 kV at at Electron microscopy unit, Faculty of Science, Mansoura University, Mansoura, Egypt.

#### Molecular identification of the selected strain and phylogenetic analysis

Genomic DNA of the strain was extracted using G-spin DNA extraction kit. The polymerase chain reaction (PCR) was performed using a reaction mixture of 1.5 μL DNA, 12.5 μL Master Mix and 0.75 μL of each of the primers and water was added up to 25 μL. The primers used were as follow: universal forward primer (8F) “AGAGTTTGATCATGGCTCAG” and universal reverse primer (1492R) “GGTTACCTTGTTACGACTT”.

PCR amplification was carried out in thermal cycles with the following PCR programme: five minutes at 95 °C as the first step, then 35 cycles were done as follows: 2 min at 95 °C for denaturation, annealing of 1 min at 48 °C, 4 min at 72 °C for elongation, and then 20 min at 72 °C for final extension. The products were kept at 4 °C. The resulting PCR product was sent to Macrogen company, South Korea, for 16S rRNA sequencing. Forward and reverse sequences were subjected to alignment via basic alignment search tool of Clustal W alignment (http://www.ebi.ac.uk/Tools/clustalw2) and the combined sequence was compared with related sequences from database of GenBank (http://blast.ncbi.nlm.nih.gov/Blast.cgi). The phylogenetic tree showing relationship of the selected strain to other *Bacillus* strains was then constructed using the Neighbor-Joining method^34^. The percentage of replicating trees in which the associated taxa clustered together in the bootstrap test (1000 replicates) using pairwise deletion option are shown next to the branches^35^. Evolutionary analyses were conducted in MEGA X ^36^.

#### Enzyme production and preparation of the crude enzyme

The strain Fatma/1 was cultured in a 250 mL Erlenmeyer flask containing freshly prepared growth medium (mentioned earlier) adjusted at pH 7. The inoculated flasks were then incubated in an incubator shaker at 120 rpm and 37 °C and allowed to grow overnight. The cultured broth was then centrifuged at 14.000 × *g* for 15 min to eliminate microbial cells and any remnants. The remaining supernatant was transferred to a clean container and stored as a crude enzyme.

#### Optimization of culture conditions for endoglucanase activity

Different pH, temperature and incubation periods were assessed for their effects on endoglucanase production. The effect of the pH of growth medium on enzyme activity was investigated by measuring the endoglucanase activity at different pH values (5–9). The isolate grown on CMC broth was incubated at a temperature range (30–60 °C) to detect the optimum temperature for the enzyme production. The effect of incubation period on the production of endoglucanase was investigated by culturing the bacteria for different incubation periods (24–96 h) at 37 °C. Selected bacterial isolate was cultured and incubated in an incubator shaker at 120 rpm and endoglucanase activity was measured at varied parameters.

#### Application of Plackett–Burman design to assess the significance and effect of process variables on endoglucanase production

Screening of important parameters that enhance the endoglucanase production was done by employing a Plackett–Burman design. Seven factors were screened, and each factor examined at two levels: − 1 for low level and + 1 for high level^37^. The set medium components investigated by the design were as follow CMC, yeast extract, peptone, K_2_HPO_4_, MnCl_2_.4H_2_O, MgSO_4_.7H_2_O and FeSO_4_.7H_2_O. Twenty runs were conducted to evaluate the effect of the selected variables on endoglucanase yield. All experiments were carried out in duplicate, and the averages of the endoglucanase activity obtained were taken as the response Table 1.

**Table 1:** Plackett–Burman experimental design matrix with the observed endoglucanase activity as affected by the seven independent variables.

Std	Run	A	B	C	D	E	F	G	Endoglucanase activity (U/mL)		Residuals
									Actual	Predicted	
17	1	1	− 1	− 1	− 1	− 1	1	− 1	14	14.66	− 0.66
16	2	− 1	− 1	− 1	− 1	1	− 1	1	0.3	− 0.92	1.22
9	3	− 1	1	1	1	1	− 1	− 1	7.2	5.86	1.34
1	4	1	− 1	1	1	− 1	− 1	− 1	20.3	20.57	− 0.27
15	5	− 1	− 1	− 1	1	− 1	1	− 1	0.4	− 0.6	1
8	6	1	1	1	1	− 1	− 1	1	22.7	23.23	− 0.53
2	7	1	1	− 1	1	1	− 1	− 1	19.9	18.76	1.14
3	8	− 1	1	1	− 1	1	1	− 1	2.3	3.28	− 0.98
10	9	1	− 1	1	1	1	1	− 1	18.5	17.91	0.59
20	10	− 1	− 1	− 1	− 1	− 1	− 1	− 1	0.5	0.04	0.46
19	11	− 1	1	1	− 1	− 1	− 1	− 1	4.4	5.94	− 1.54
6	12	1	1	− 1	− 1	1	1	− 1	15.1	16.18	− 1.08
5	13	1	− 1	− 1	1	1	− 1	1	14.3	16.28	− 1.98
12	14	1	− 1	1	− 1	1	1	1	17.7	17.03	0.67
14	15	− 1	− 1	1	− 1	1	− 1	1	2.2	2.41	− 0.21
11	16	− 1	1	− 1	1	1	1	1	0.3	1.01	− 0.71
18	17	1	1	− 1	− 1	− 1	− 1	1	19.3	18.93	0.37
7	18	1	1	1	− 1	− 1	1	1	22.4	20.65	1.75
13	19	− 1	1	− 1	1	− 1	1	1	2.3	2.06	0.24
4	20	− 1	− 1	1	1	− 1	1	1	2	2.82	− 0.82

Variable level	g/L	g/L	g/L	g/L	g/L	g/L	g/L				
− 1	2	2	1	0.5	0.05	0.05	0.01				
1	18	8	9	2	0.45	0.45	0.1				

The independent factors are: A (CMC), B (Peptone), C (Yeast extract), D (K_2_HPO_4_), E (MgSO_4_·7H_2_O), F (FeSO_4_·7H_2_O), G (MnCl_2_·4H_2_O).

Plackett–Burman experimental design is based on the following equation of the first order polynomial model:

2 $$ Y = {\beta_0} + \sum\limits {{\beta_i}{X_i}} $$

where Y is the response (endoglucanase activity), β_0_ is the intercept of the model, β_i_ is the linear coefficient, and X_i_ is the level of the independent factors.

#### Optimization of endoglucanase production by response surface methodology

The face centered central composite design (FCCCD) was employed to determine the optimum levels of four significant variables (CMC, peptone, yeast extract and FeSO_4_.7H_2_O) and to assay the individual and mutual interactions within these variables on the production of endoglucanase. A total of 30 trials were carried out in the FCCCD which is a statistical experimental design with each factor being varied on three different levels, high (+ 1), medium (0), low (− 1). The second-degree polynomial equation was employed to figure out the relationship between the independent variables and endoglucanase production. Given all linear, square and interaction coefficients, the quadratic regression model can be explained as follows:

3 $$Y = {\beta_0} + \sum\limits_i {{\beta_i}{X_i} + \sum\limits_{ii} {{\beta_{ii}}{X_i}^2} } + \sum\limits_{ij} {{\beta_{ij}}{X_i}{X_j}} $$

In which "Y is the predicted response, β_0_ is the regression coefficients, β_i_ is the linear coefficient, β_ii_ is the quadratic coefficients, β_ij_ is the interaction coefficients, and X_i_ is the coded levels of independent variables".

### Statistical analysis

The regression analysis of the results was carried out to calculate the analysis of variance. Minitab software (version 19) was used to design the experiment and for subsequent statistical analysis of Plackett–Burman experiment. The experimental designs and statistical analysis for FCCCD were executed by Design Expert version 12 for Windows software. The statistical software package, STATISTICA software (Version 8.0, StatSoft Inc., Tulsa, USA) was used to plot the three-dimensional surface graphs.

### Enzyme assay and protein estimation

Endoglucanase activity was measured using carboxymethyl cellulose as substrate. A solution of CMC (1% w/v) was prepared by dissolving CMC powder in 0.05 M Na-citrate buffer (pH 5.0) on a stirrer and heater at 60 °C for 1 h. After that the solution was centrifuged at 10,000 × *g* for 10 min to get a clear solution. A 900 μL of the prepared substrate were mixed with 100 μL of the crude endoglucanase sample. The mixture was incubated at 50 °C. After 10 min, 1.5 mL of dinitrosalicylic acid (DNS) was added to each tube and the mixture was allowed to boil for 5 min then cooled in ice. The amount of reducing sugars produced were measured at 540 nm. A standard curve is produced using known concentrations of glucose plotted against A540. One unit (U) of endoglucanase activity is defined as the amount of endoglucanase required to produce 1 μmol of reducing sugar in one minute under the assay conditions^38^. Protein concentration of samples was obtained using Bradford method with bovine serum albumin as a standard^39^.

### Purification of endoglucanase

The crude enzyme was precipitated using solid ammonium sulfate saturation (40–80%). Ammonium sulfate was added gradually to the crude enzyme in an ice bath. Flasks were kept on a magnetic stirrer at 4 °C for 12 h before centrifugation at 9000 × *g* for 15 min. A 50 mM sodium phosphate buffer saline (pH 7.0) was used to dissolve the pellet obtained via centrifugation. The dialysis of the ammonium sulfate was performed in a pre-treated dialysis tube (SERVA pro, 44144, diameter 21 mm × 5 m). Precipitate produced during dialysis was collected by centrifugation and was eliminated. Dialyzed samples were subjected to endoglucanase activity assay and protein estimation via the Bradford method^39^ and kept at 4 °C for subsequent purification steps.

The dialyzed sample was further purified in DEAE-Sepharose CL6B column previously equilibrated using phosphate buffer pH 7.0. The column was washed twice with the same buffer solution and the protein adsorbed on the column was then eluted using a gradient of 0–1.0 M NaCl in phosphate buffer at a flow rate of 1 mL/min. Fractions of 3 mL each were collected. Endoglucanase activity assay and protein estimation via the Bradford method^39^ were carried out to determine the fractions with the highest endoglucanase activity which were then pooled.

### Enzyme molecular weight determination

The molecular weight of the enzyme was determined through SDS-PAGE (sodium dodecyl sulfate polyacrylamide gel electrophoresis) using the method of Laemmli^40^. A 10% (w/v) separating gel and 5% (w/v) stacking gel with 0.1% (w/v) SDS were prepared. Markers of standard proteins with known molecular weights were loaded next to the purified enzyme protein and Coomassie brilliant blue R-250 was used to stain the gel, then the destaining was done using a solution of methanol, acetic acid and water in the ratio of 4:1:5 to visualize the protein bands.

### Physicochemical characterization of endoglucanase

Endoglucanase activity was measured at different pH values by dissolving carboxymethyl cellulose as a substrate in different buffers of 50 mM Na-citrate buffer (3–6), sodium phosphate buffer (7–8), Tris–HCl (pH 8–9) and glycine–NaOH buffer (9–10.5). The optimum temperature for endoglucanase activity was determined by incubating the assay mixture at different temperatures (25 to 60 °C) in sodium phosphate buffer under the assay conditions. The effect of incubation time on enzyme activity was assessed by incubating the reaction mixture for different times (from 10 to 90 min). Enzyme activity was assayed through the DNS method as reported earlier.

### Thermal stability of endoglucanase

The effect of temperature on the stability of endoglucanase was determined by incubating the enzyme for different times (from 10 to 90 min) at 40, 50, 60, 70 and 80 °C prior to addition of the substrate. The enzyme was then cooled on ice and the residual activity was measured.

### Estimation of deactivation rate constant (k_d_) and half-life time (T_1/2_)

The heat inactivation half-life (T_1/2_) and thermal deactivation constant (k_d_) of the endoglucanase produced by *Bacillus subtilis* strain Fatma/1 were calculated via GraphPad Prism 5 software (GraphPad Software Inc., San Diego, CA).

The half-life of the enzyme activity (T_1/2_) is the period required for the enzyme activity to decrease to a half of its initial activity. The half-life time was determined by using the following equation:

4 $$ {T_{1/2}} = \frac{\ln 2}{{k_d}} = \frac{0.693}{{k_d}}$$

where: K_d_ is deactivation rate constant.

### Effect of pH on endoglucanase stability

The optimum pH for endoglucanase stability was determined through pre-incubation of the enzyme in absence of its substrate at room temperature for 1, 6, 18, and 24 h in buffers of varied pH values (3–10). The residual activity was measured under the standard assay conditions.

### Isolation and identification of the bacterial pathogens

Biofilm forming bacterial strains were isolated from diabetic foot patients admitted to Menof fever hospital, Menofia, Egypt. One strain of each of the following: *Pseudomonas aeruginosa* (Pa)*, Staphylococcus aureus* (Sa) were used in this study. *Pseudomonas aeruginosa* was isolated on MacConkey agar while *Staphylococcus aureus* was isolated on Mannitol salt agar. All isolates were preserved in 20% glycerol 80% nutrient broth at -18 °C. Isolated pathogens were identified by a pre-established series of rapid biochemical conventional tests.

### In vitro biofilm formation

For biofilm formation in vitro, 200 μL of brain heart infusion broth (BHI) were aseptically introduced in 1.5 mL microtubes. 10 µL of suspensions containing 1 × 10^6^ CFU/mL of each strain were prepared and added separately into the microtubes which were then incubated at 37 °C for 24 h.

### Tube method

A slightly modified tube method (TM) of Christensen et al.^41^ that is a qualitative assay for detection of biofilm producer microorganism was used**.** Isolates are inoculated in test tube which contained brain heart infusion broth (BHI) and incubated for 24 h at 37 °C. Endoglucanase at a concentration of 12 U/mL was added to the tubes and left for one hour. Control tubes with no enzyme treatment were also set for comparison. The isolates, of which biofilms formed on the walls of test tube are stained with safranine for 1 h. Then, safranine-stained test tube is rinsed twice with phosphate-buffered saline (PBS) to discharge stain. The tubes were allowed to air-drying and the occurrence of visible film lined the walls of the tube indicates biofilm production.

### Enzymatic treatment of different species biofilms

The effect of the endoglucanase against the biofilm forming strains was tested on 96-well polystyrene plates through the method of Trivedi et al.^42^. Endoglucanase at concentrations (4, 8, 12 U/mL) were added in BHI containing the bacterial suspension of overnight culture (10^6^ CFU/mL) of each strain. The plates were then incubated for 24 h at 37 °C. After incubation, the planktonic cells and media were disposed, and weakly adherent cells were rinsed off by washing with deionized water and left to air dry before staining. The biofilms were stained by 400 μl of 0.4% (w/v) crystal violet solution for 10 min. The dye was then discarded, and the wells were rinsed twice with deionized water. The plates were allowed to dry before 1 mL of absolute ethanol was used to solubilize the dye. The optical density was measured at 600 nm in Stat Fax-2100 plate reader.

### Microscopic observation of biofilm

For visualization of biofilms by light microscopy, the biofilms of each strain were grown for 24 h at 37 °C on glass pieces (1 × 1 cm size) placed in 96-well polystyrene plates filled with growth media. Endoglucanase was added after 24 h of growth. Slides were stained using the Gram staining technique. Stained glass pieces were placed on slides and examined by light microscopy. Visible biofilms were documented with an attached camera.

### Bactericidal effect of endoglucanase on planktonic cells

The effects of endoglucanase on planktonic bacterial growth were compared with the effects on the biofilms through zone of inhibition study on Mueller Hinton agar plates. This was done using different concentrations of endoglucanase (4–8–12 U/mL).

### Estimation of carbohydrate content of the biofilm

Extracellular polymeric substance was extracted through formaldehyde-NaOH method^43^. One mL of 10% formaldehyde solution was added to the components of each well and kept at 4 °C for 1 h. Formaldehyde solution was then discarded and 1 mL of 1 M NaOH solution was added and kept at 4 °C for 2 h under shaking conditions. The suspension of each treatment was collected and filtered through 0.22 micron filter. The filtrate was dialyzed against distilled water. Biofilm`s total carbohydrate content was determined through phenol–sulfuric method with glucose as a standard^44^.

## Results and discussion

### Isolation and screening of cellulolytic bacteria

**Figure 1:**
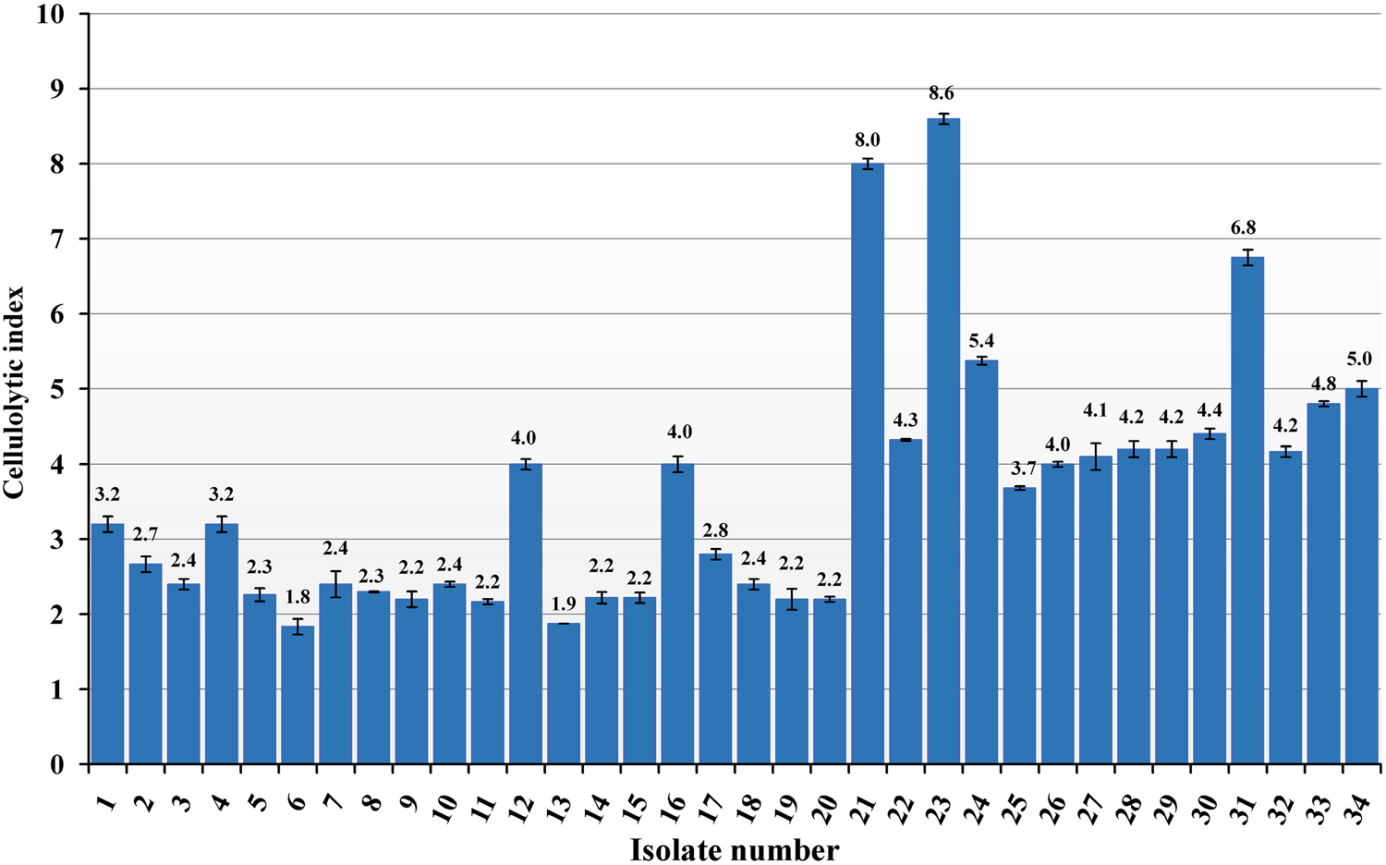
Cellulolytic index of different isolated bacterial strains. The mean difference is significant at 0.01 level.

In this study, thirty-four bacterial strains were isolated from the soil and examined for their potential for endoglucanase productivity on carboxymethyl cellulose (CMC) agar medium to select the potential isolates showing best cellulolytic index. The average of the ratio is given in Fig. 1. Among the 34 isolates, the isolate number 23 showing the best cellulolytic index was selected for further secondary screening (Fig. 1). The isolate was named Fatma/1.

### Biochemical, microscopic examination and molecular phylogenetic analysis using 16S rRNA gene sequence

**Figure 2:**
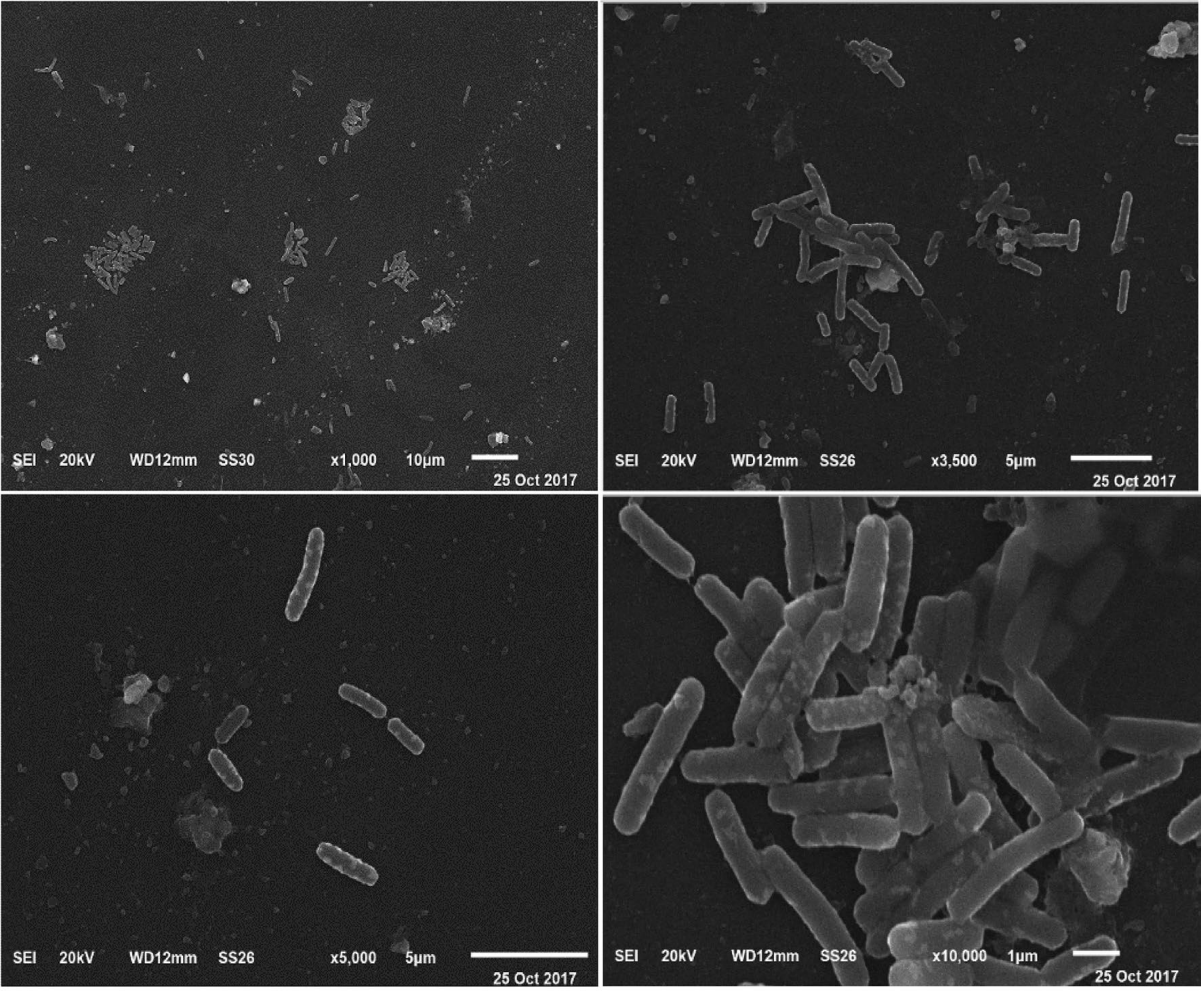
SEM image of Bacillus subtilis strain Fatma/1 at different magnifications.

**Figure 3:**
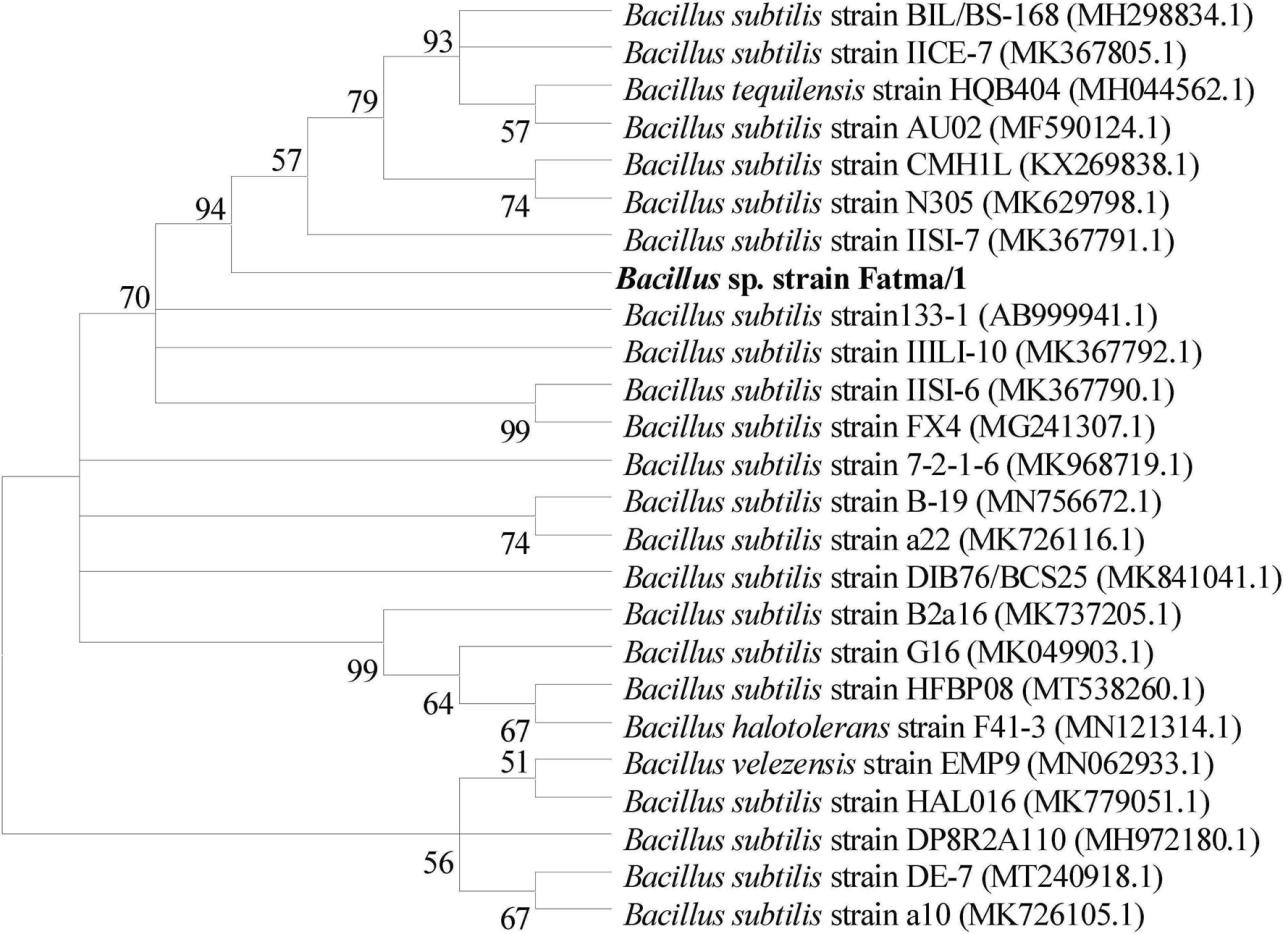
Phylogenetic tree showing the relationship between bacterial isolate Fatma/1 and other *Bacillus* strains.

The selected strain was examined microscopically revealing a gram-positive bacteria with a rod shape cell (Fig. 2). The isolate was identified as *Bacillus subtilis*. The bacterial isolate was identified according to the Bergey's Manual of Systematic Bacteriology^45^. In order to confirm the identification, the isolate was further analyzed by 16S rRNA sequencing and identified as *Bacillus subtilis* strain Fatma/1. The sequence was submitted to GenBank under accession number LC535007.1. Figure 3 reveals phylogenetic tree showing the relationship between the bacterial strain Fatma/1 and other *Bacillus* strains.

### Effect of different culture conditions on endoglucanase activity

Bacterial growth and enzyme production are highly influenced by the chemical composition of the growth media and by the environmental factors. Every strain has a distinct physicochemical and dietary needs for development and enzyme secretion^11^. Different production parameters were optimized. Fixed agitation speed (120 rpm) was used during this study. Rastogi et al*.*^46^ and Deka et al.^47^ found that an agitation speed of 121 rpm is the optimum speed for endoglucanase production by *Bacillus subtilis*.

### The effect of temperature on endoglucanase production

**Figure 4:**
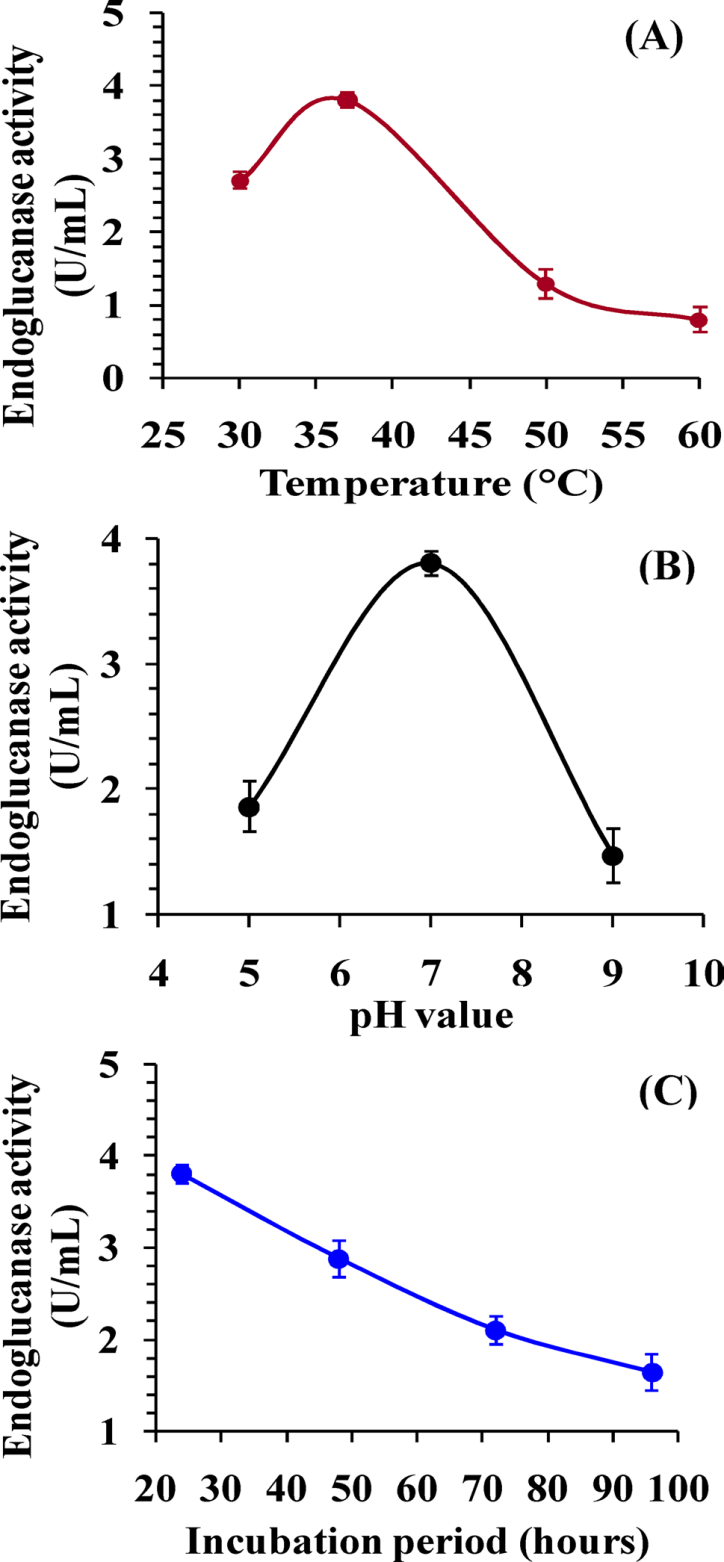
Effect of temperature (**A**), pH (**B**), and incubation period (**C**) on endoglucanase production.

Temperature is a very important parameter that affects the activity of the enzyme and is crucial for a fermentation process^46^. The medium temperature affects extracellular enzyme production through alteration of the physical properties of cell membrane^48^. *Bacillus subtilis* strain Fatma/1 used in this study yielded maximum endoglucanase production at 37 °C as shown in Fig. 4A. These results are in agreement with other studies performed on cellulase production by *Bacillus* spp. Optimum temperature for production of cellulase by *B. amyloliquefaciens* DL-3^49^, *Bacillus pumilus* EB3^50^ and *Bacillus amyloliquefaciens*^51^ was 37 °C. Islam et al.^52^ reported maximum enzyme production by *Bacillus* sp. at 35 °C.While Deka et al.^47^ confirmed that 39 °C is the ideal temperature for cellulase production by *Bacillus subtilis*. At temperatures lower than the optimum, transport of substances over the cells is inhibited and lower amount of enzymes are obtained^53^. At temperatures above the optimum, the enzyme production gradually decreased due to denaturation and the conformation change of the enzyme, as enzymes are proteins^13,21^.

### The effect of pH on endoglucanase activity

The pH of the growth medium was found to be an important parameter as it affects chemical reactions by regulating the movement of reactants and enzymes through the cell membrane^54^. Heck et al.^55^ stated that control of pH during fermentation was necessary for improved productivity of cellulase. The optimum pH of the isolated organism shown at Fig. 4B was similar to the optimal pH values of most of the *Bacillus* spp*.*^56^. Many workers reported significant hydrolysis of cellulose due to enzyme activity at pH ranging from 6.5 to 7.5^57^. While Özmen^58^ reported 8.0 to be the ideal pH for production of cellulase by *Bacillus subtilis*. The effect of pH on enzyme activity may be attributed to the denaturation of enzyme proteins that takes place at pH varying from the enzyme`s optimum pH value^59^. The effect of the pH factor on enzyme yield is also attributed to the effects on cell permeability and the stability of the enzyme released^57^. Providing optimal pH conditions can stimulate strain growth, increase cellulase yield and maintain the enzyme's negative feedback mechanism^60^.

### The effect of incubation period on endoglucanase activity

Figure 4C indicated that the maximum cellulolytic activities were obtained at 24 h of incubation period, which agrees with the results obtained by Sreena et al.^61^ with the same species. Similar results were reported for *Bacillus* sp.^52,55,62^ and *Paenibacillus* sp.^63^. However, Rathnan et al.^64^ reported major peak of activity at 60 h of incubation. Goyal et al.^65^ reported maximum CMCase activity at 60 h using static conditions and at 48 h if the bacteria was incubated in a shaker. This indicates that shaking reduces the maximum time required for cellulose production. The decrease in enzyme activity after reaching a certain point could be due to catabolic repression of byproducts of cellulase (e.g. glucose) action on CMC which inhibit enzyme activity^21^. Consumption of nutrients results in starvation, decrease in bacterial growth and formation of dormant spores and CMCase activity is directly related to growth rate as revealed by Yang et al.^60^.

### Application of Plackett–Burman design to assess the significance and effect of process variables on endoglucanase production

A 20 run experimental design was performed to evaluate the impacts of seven medium components on the endoglucanase production. The obtained results showed a wide variation (0.3 to 22.7 U/mL) in endoglucanase production. This marked variation elucidated that the optimization process was necessary to achieve maximum endoglucanase production (Table 1). Data in Table 1 presents the predicted endoglucanase activity versus the actual activity which confirms the model`s adequacy and reveals a similarity among the experimental findings and the values given by the model.

The model yielded maximum endoglucanase activity of 22.7 U/mL in trial number 6 displaying significant increment compared to trial number 2 that yielded an activity of only 0.3 U/mL. This was obtained at pH 7.0 under 24 h of the production period at 37 °C. A large *T*-value associated with a little *P*-value implies that the corresponding model term is of high significance. The positive values of linear coefficients associated with some factors indicate that endoglucanase enzyme increased with an initial increase of such factors, while negative coefficients represent factors that caused decline in endoglucanase production. Among different medium components investigated, CMC, yeast extract, peptone, K_2_HPO_4_, and MnCl_2_·4H_2_O exhibited a positive impact on endoglucanase production, whereas FeSO_4_·7H_2_O and MgSO_4_·7H_2_O had a quite a negative impact. Ahmad et al*.*^66^ also reported a negative effect of FeSO_4_ on the enzyme activity. This may be attributed to the inhibition of endoglucanase production by metal ions and potential use of sulfate in the protein assembly^67^. While Ye et al*.*^51^ showed that addition of Fe^2+^ to the growth medium enhanced the activity of cellulase by 68.5% till certain level above which enzyme activity diminished.

The chemical components of the growth medium greatly affect cell growth and cellulase production. CMC was used as the sole source of carbon in this experiment. CMC showed to be an effective inducer for producing cellulase^68^. Islam and Roy^63^ showed that among different substrates used in their study, CMC was the optimal carbon source for growth and endoglucanase production by *Bacillus* species.

Organic sources of nitrogen were reported to be more appropriate for the production of cellulase by *Bacillus* species than inorganic sources^31^. Yang et al.^60^ explained the importance of using organic nitrogen sources with *Bacillus subtilis* to reveal enzyme production and observed maximum CMCase activity when equal amounts of peptone and yeast extract were used as the only nitrogen supply. Kaur and Joshi^20^ reported the efficiency of yeast extract and peptone in enhancing endoglucanase production by *Penicillium chrysogenum* and *Trichoderma reesei.* Yeast extract is considered a cheap organic source of vitamins, vital amino acids and proteins as revealed by Sahlan et al.^69^. El-Naggar et al*.*^13^ reported peptone as the optimum supply of nitrogen for CMCase production followed by yeast extract. When using inorganic nitrogen forms (urea or NH_4_NO_3_) as the only supply of nitrogen element, Yang et al*.*^60^ reported almost negligible cellulase activity since the metabolism of mineral nitrogen causes the medium to be acidified, hence generating a negative effect on enzyme production.

Phosphate (K_2_HPO_4_) has an important role in bacterial growth and metabolism. It acts an effector of many enzymatic reactions in primary metabolism, such as respiration, nucleic acid and protein synthesis, and control levels of adenosine triphosphate^70^.

Bakare et al.^71^ emphasized the important role of Mg^2+^ ions in increasing and stabilizing the process of cellulase production. Gomaa^70^ reported the inhibitory effect of MgSO_4_ on CMCase activity when added in a concentration above 0.4 g/L. Sreena and Sebastian^11^ stated the significance of MgSO_4_ at its lower concentration (0.01 g/L) as it plays a crucial role in initial cell growth.

The variables are considered crucial when having confidence levels higher than 95%. CMC and yeast extract were markedly significant at 100% confidence level for endoglucanase production, whereas peptone was found significant at the 99.9% level for endoglucanase production. However, FeSO_4_·7H_2_O indicated significance of 98.3%. Other coefficient terms used within this model did not really have notable significance for endoglucanase production. The contribution percentages for each parameter used within the model are shown in Table 2. This suggests that increasing the amounts of CMC, peptone, yeast extract in growth medium along with decreasing FeSO_4_·7H_2_O levels should greatly enhance endoglucanase production.

**Table 2:** Plackett–Burman design regression analysis with the main effect, contribution (%), coefficients estimate, *T*-value and confidence level for the model and the seven independent variables.

Term	Coefficient estimate	Main effect	Contribution (%)	*T*-value	Confidence level
Intercept	10.305	‒	‒	35.45	100
A-CMC	8.115	16.23	85.346	27.92	100
B-Peptone	1.285	2.57	5.068	4.42	99.9
C-Yeast extract	1.665	3.33	8.755	5.73	100
D-K_2_HPO_4_	0.485	0.97	0.479	1.67	87.9
E-MgSO_4_·7H_2_O	− 0.525	− 1.05	0.138	− 1.81	90.4
F-FeSO_4_·7H_2_O	− 0.805	− 1.61	0.212	− 2.77	98.3
G-MnCl_2_·4H_2_O	0.045	0.09	0.003	0.15	12
R^2^	98.60%				
Adj R^2^	97.78%				
Pred R^2^	96.11%				

The reliability of fit can, however, be further assured by the coefficient of determination (R^2^). The R-squared is always between 0 and 100. The greater R^2^ value reflected adequately fits between the observed and predicted responses and explains the variation in the response variable around its mean^72^. The correlation coefficient (R^2^) generated by the model was observed to be 98.6%. This means that 98.6% of experimental data of the model were compatible with only 1.4% variation that could not be explained by the model (Table 2). Moreover, the great values of predicted R^2^ (96.11%) and adjusted R^2^ (97.78%) indicated a strong correlation between theoretical and experimental values.

The ANOVA results generated by the model are presented in Table 3. By employing the regression analysis, the variables which showed to be significant at or above the 95% level (*P*-value < 0.05), were believed to have greater influence on endoglucanase activity. The high Fisher’s *F-*test value of the model (120.79) with the very low *P*-values (*P* = 0) implies that the model is significant. Factors with probability values lower than 0.05 is said to enhance endoglucanase production. A particular variable is said to be highly significant when having a high *T*-test value associated with a low *P*-value^11^. CMC and yeast extract have *P*-value of 0. While peptone have a *P-*value of 0.001 (Table 3). This indicates high significance of these factors for maximum production of endoglucanase. The negative coefficient obtained for FeSO_4_·7H_2_O with a *P*-value of 0.017 (less than 0.05) confirms the negative effect of this factor on the productivity of endoglucanase.

**Table 3:** Analysis of variance of endoglucanase activity versus the seven independent variables.

Source	DF	Adj SS	Adj MS	*F*-Value	*P*-Value
Model	7	1428.75	204.11	120.79	0
Linear	7	1428.75	204.11	120.79	0
A-CMC	1	1317.06	1317.06	779.4	0*
B-Peptone	1	33.02	33.02	19.54	0.001*
C-Yeast extract	1	55.44	55.44	32.81	0*
D-K_2_HPO_4_	1	4.7	4.7	2.78	0.121
E-MgSO_4_·7H_2_O	1	5.51	5.51	3.26	0.096
F-FeSO_4_·7H_2_O	1	12.96	12.96	7.67	0.017*
G-MnCl_2_·4H_2_O	1	0.04	0.04	0.02	0.88
Error	12	20.28	1.69		
Total	19	1449.03			

*Significant values, *df* Degree of freedom, *F* Fishers’ function.

**Figure 5:**
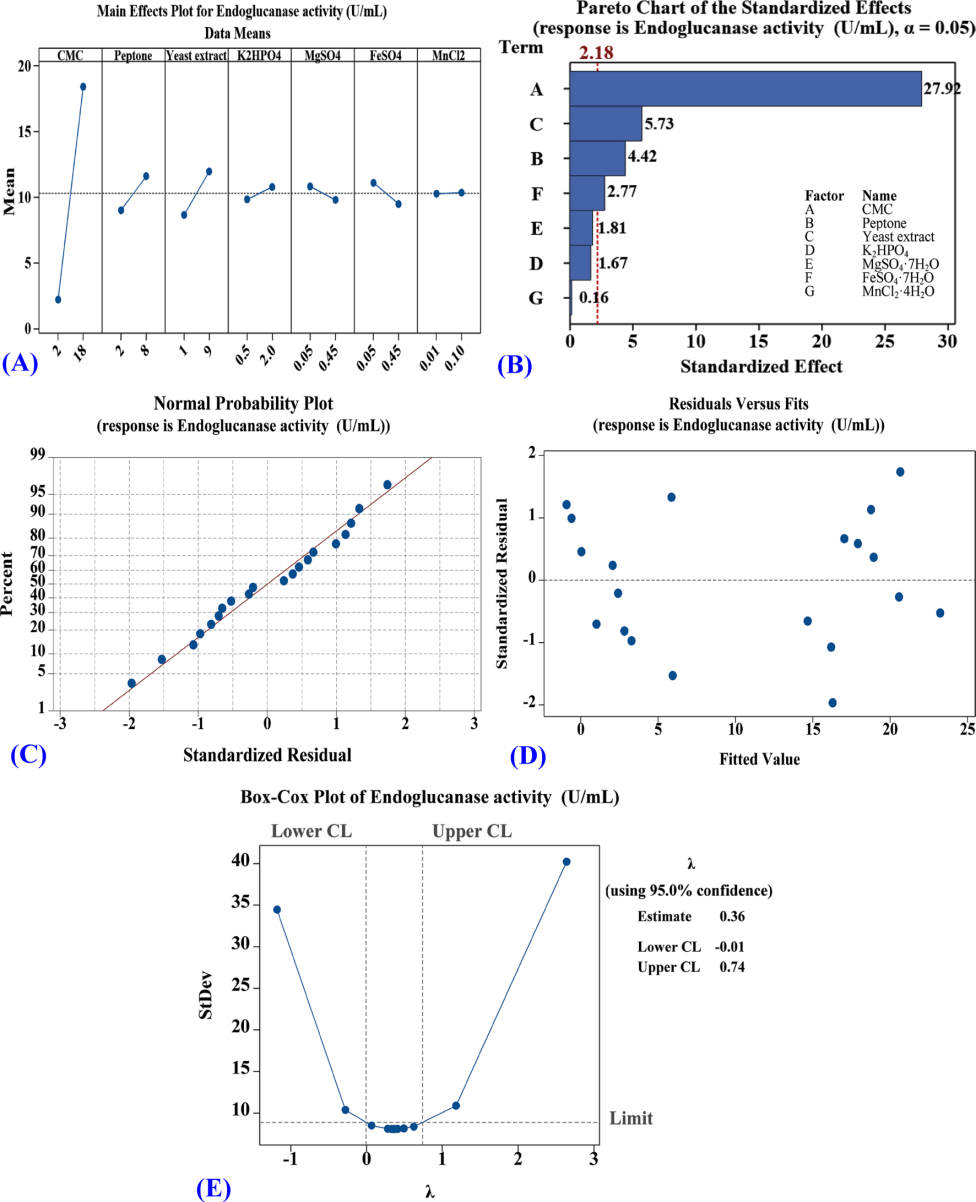
Plackett-Burman design for optimization of endoglucanase production by *Bacillus* subtilis Fatma/1. **A**) Main Effects Plot of the seven independent variables on the mean endoglucanase activity, **B**) Pareto chart illustrates the order and significance of the variables, **C**) Normal Probability Plot, **D**) Residuals vs Fits and **E**) Box-Cox plot.

By plotting the value of the mean of endoglucanase activity at each level of the seven independent variables, a main effects plot is produced (Fig. 5A). These mean values are similar to those displayed in Table 2. The plot reveals the most important factors that should maximize endoglucanase productivity. The more the line is not parallel to the x-axis, the greater the strength of the main effect. Horizontal line means the main effect does not exist. Data in Fig. 5A showed that all factors have varying effects on enzyme production except MnCl_2_·4H_2_O illustrated with a horizontal line.

A Pareto chart (Fig. 5B) indicates the impact of the various parameters investigated. The height of each bar is proportional to the value of its estimated impact. The bars with the greatest effects are displayed on top. This graph is commonly used in quality control settings to detect critical factors that lead to defects in a procedure^73^. Pareto chart Fig. 5B revealed that CMC (A) was the best factor affecting endoglucanase production and hence activity (27.92) followed by Yeast extract (C), peptone (B), FeSO_4_·7H_2_O (F), MgSO_4_.7H_2_O (E), K_2_HPO_4_ (D), then MnCl_2_·4H_2_O (G).

The normal probability plot (NPP) is shown in Fig. 5C. It is an important graphical technique to test the adequacy of the model^74^. By plotting the residuals against the theoretical values predicted by the model, the residuals should fall in close proximity to the straight line representing the desired distribution. In the present model, the NPP plot reveals that many residuals from the fitted model are in close proximity to the reference line. This indicates that the model successfully suited the experimental results.

Furthermore, the residual analysis (Fig. 5D) obtained by plotting of the observed–predicted values (residuals) *vs* the response (observed endoglucanase activity) indicated that the residuals were symmetrical and uniformly spread all through the range.

The Box-Cox plot for endoglucanase production is shown in Fig. 5E. It revealed that the normal distribution can be achieved using Lambda values between − 0.01 and 0.74. Transformation is not required for the model used for endoglucanase production as lambda value (0.36) falls between − 0.01 and 0.74.

### Regression equation

On application of ANOVA, it was found that the first order model describing the correlation between the 7 parameters investigated through 20 runs and the endoglucanase activity could be presented as the equation:

5 $$ {\text{Endoglucanase}} \, {\text{activity}} \, \left( {{{\text{U/mL}}}} \right) \, = 10.305 \, + \, 8.115 \, {\text{CMC}} \, + \, 1.285 \, {\text{peptone}} \, + \, 1.665 \,{\text{ yeast}} \, {\text{extract}} \, + \, 0.485 \, {{\text{K}}_2}{\text{HP}}{{\text{O}}_4} - \, 0.525 \, {\text{MgS}}{{\text{O}}_4}.7{{\text{H}}_2}{\text{O}} \, - \, 0.805 \, {\text{FeS}}{{\text{O}}_4}\cdot7{{\text{H}}_2}{\text{O}} \, + \, 0.045 \, {\text{MnC}}{{\text{l}}_2}.4{{\text{H}}_2}{\text{O}}$$

Maximum endoglucanase yield was obtained at run 6 with the following set: 18 g/L CMC, 8 g/L peptone, 9 g/L yeast extract, 2 g/L K_2_HPO_4_, 0.05 g/L MgSO_4_, 0.05 g/L FeSO_4_ and 0.01 g/L of MnCl_2_.

### Statistical optimization of endoglucanase enzyme production by *Bacillus subtilis* strain Fatma/1 using face centered central composite design (FCCCD)

Placket-Burman design results revealed that, CMC (X_1_), peptone (X_2_), yeast extract (X_3_) and Fe_2_SO_4_·7H_2_O (X_4_) were the most significant factors to produce endoglucanase by *Bacillus subtilis* strain Fatma/1. These four factors were selected for further optimization using face centered central composite design. The four factors employed, and their coded and actual levels used in this design are shown in Table 4. Variables with a positive effect were employed at higher levels. Insignificant variables determined by Placket-Burman design results were maintained in all trials at their low levels.

**Table 4:** Face-centered central composite design matrix representing endoglucanase production by *Bacillus subtilis* strain Fatma/1 as affected by CMC, yeast extract, peptone and FeSO_4_ concentrations with coded and actual factor levels.

Std	Run	Variables				Endoglucanase production (U/mL)		Residuals
		X_1_	X_2_	X_3_	X_4_	Actual	Predicted	
27	1	0	0	0	0	28.99	29.60	− 0.61
13	2	− 1	− 1	1	1	17.42	17.56	− 0.14
7	3	− 1	1	1	− 1	20.02	20.11	− 0.09
3	4	− 1	1	− 1	− 1	22.88	23.25	− 0.37
18	5	1	0	0	0	21.53	21.60	− 0.07
4	6	1	1	− 1	− 1	20.15	19.76	0.39
9	7	− 1	− 1	− 1	1	19.32	19.70	− 0.38
17	8	− 1	0	0	0	25.48	24.98	0.50
21	9	0	0	− 1	0	32.37	31.82	0.55
15	10	− 1	1	1	1	19.79	19.75	0.04
1	11	− 1	− 1	− 1	− 1	22.10	21.98	0.12
12	12	1	1	− 1	1	19.24	19.70	− 0.46
23	13	0	0	0	− 1	29.25	29.08	0.17
25	14	0	0	0	0	29.51	29.60	− 0.09
11	15	− 1	1	− 1	1	22.23	22.02	0.21
29	16	0	0	0	0	29.64	29.60	0.04
28	17	0	0	0	0	29.25	29.60	− 0.35
16	18	1	1	1	1	16.64	16.51	0.13
22	19	0	0	1	0	28.60	28.72	− 0.12
6	20	1	− 1	1	− 1	14.56	14.53	0.03
19	21	0	− 1	0	0	24.70	24.39	0.31
10	22	1	− 1	− 1	1	17.68	17.35	0.33
30	23	0	0	0	0	29.18	29.60	− 0.42
2	24	1	− 1	− 1	− 1	18.07	18.46	− 0.39
5	25	− 1	− 1	1	− 1	19.08	18.97	0.11
26	26	0	0	0	0	29.77	29.60	0.17
8	27	1	1	1	− 1	15.73	15.70	0.03
14	28	1	− 1	1	1	14.30	14.29	0.01
20	29	0	1	0	0	26.26	26.14	0.12
24	30	0	0	0	1	28.60	28.34	0.26

Variable (g/L)	Code code	Coded and actual levels						
		− 1	0	1				
CMC conc	X_1_	12	18	24				
Peptone conc	X_2_	4	8	12				
Yeast extract conc	X_3_	7	9	11				
FeSO_4_·7H_2_O conc	X_4_	0.05	0.1	0.15				

Experimental and predicted endoglucanase activities for the thirty trials of the employed design matrix are illustrated in Table 4. The results show considerable variation in the endoglucanase activity based on the varying four independent variables.

Based on the obtained experimental data; endoglucanase activity ranged from 14.3 to 32.37 U/mL. The highest yield of endoglucanase (32.37 U/mL) was obtained at run no. 9, using 18 g/L carboxymethyl cellulose, 8 g/L peptone, 7 g/L yeast extract and 0.1 g/L FeSO_4_.7H_2_O. Table 5 denoted a concise comparison between cellulase activity obtained in this study and some novel isolated *Bacillus* strains and their enzyme activities.

**Table 5:** Some novel isolated *Bacillus* strains and their enzyme activity.

*Bacillus subtilis* strain	Enzyme activity (U/mL)	References
*Bacillus subtilis* A8	50.8	Soeka^97^
*Bacillus* sp.	2.76	Islam et al.^52^
*Bacillus subtilis* AS3	0.75	Deka et al.^47^
*Bacillus subtilis* MU S	566.66	Sreena and Sebastian^11^
*Bacillus subtilis* BY-2	3.56	Yang et al.^60^
*Bacillus* sp.	29.8	Thakkar and Saraf^98^
*Bacillus subtilis* Fatma/1	32.37	Current study

### Multiple regression analysis and ANOVA

The FCCCD results were analyzed using multiple regression analysis and illustrated in Table 6. The correlation coefficient (R^2^) generated by the model was observed to be 0.997. This means that 99.7% of experimental data of the model were compatible with only 0.3% variation that could not be explained by the model (Table 6). A regression model having an R^2^-value higher than 0.9 was considered as having a very high correlation^75^.

**Table 6:** Analysis of variance for endoglucanase production by *Bacillus subtilis* strain Fatma/1 as affected by CMC, yeast extract, peptone and FeSO_4_ concentrations.

Source of variance		Coefficient estimate	Degrees of freedom	Sum of Squares	Mean Square	*F*-value	*P*-value
Model		29.6	1	849.96	60.71	361.94	< 0.0001*
Linear effect	X_1_	− 1.69	1	51.41	51.41	306.48	< 0.0001*
	X_2_	0.87	1	13.70	13.70	81.68	< 0.0001*
	X_3_	− 1.55	1	43.24	43.24	257.77	< 0.0001*
	X_4_	− 0.37	1	2.44	2.44	14.56	0.0017*
Interaction effect	X_1_X_2_	0.01	1	0.00	0.00	0.01	0.9254
	X_1_X_3_	− 0.23	1	0.85	0.85	5.08	0.0396*
	X_1_X_4_	0.29	1	1.37	1.37	8.16	0.012*
	X_2_X_3_	− 0.03	1	0.02	0.02	0.10	0.7553
	X_2_X_4_	0.26	1	1.11	1.11	6.61	0.0213*
	X_3_X_4_	0.22	1	0.76	0.76	4.52	0.0505
Quadratic effect	X_1_^2^	− 6.31	1	103.28	103.28	615.73	< 0.0001*
	X_2_^2^	− 4.34	1	48.75	48.75	290.64	< 0.0001*
	X_3_^2^	0.67	1	1.15	1.15	6.88	0.0192*
	X_4_^2^	− 0.89	1	2.07	2.07	12.31	0.0032*
Error effect	Lack of fit		10	2.07	0.21	2.34	0.1802
	Pure error		5	0.44	0.09		
R^2^	0.997	Std. Dev	0.41				
Adj R^2^	0.9943	Mean	23.08				
Pred R^2^	0.9852	C.V. %	1.77				
Adeq Precision	60.55						

*Significant values, *F*: Fishers's function, *P*: Level of significance, C.V: Coefficient of variation.

Moreover, the great values of predicted R^2^ (0.9852) and adjusted R^2^ (0.9943) indicated a strong correlation between theoretical and experimental values and the high significance of the model (Table 6). As well, the low value of the coefficient of variation (C.V. = 1.77%) indicates precision and adequate reliability of the experiments performed^76^.

The predicted residual sum of squares (PRESS) revealed in Table 6 determines how well the model fits each point in the design. The small PRESS value of 12.60 obtained by the design indicates that the model fits the data points well.

The ANOVA of the quadratic regression model is shown in Table 6. The high Fisher’s *F-*test value of the model (361.94) with the very low *P*-values (< 0.0001) implies that the model is significant (Table 6). The high *F*-value and nonsignificant lack of fit (0.1802) also suggested that the results obtained was in a good fit with the model.

The significance of each coefficient was determined by *P*-values as listed in Table 6. Factors with probability values lower than 0.05 is said to enhance impact on endoglucanase production. A positive or negative response is detected by positive or negative sign of the Coefficient to assay the mutual interactions between the test variables which could be synergistic (positive coefficient) or antagonistic (negative coefficient).

It is observed from the degree of significance that the linear coefficients of X1, X_2_, X_3_, X_4_, interactions between X_1_X_3_, X_1_X_4_ and X_2_X_4_, the quadratic effect of X1^2^, X_2_^2^, X_3_^2^, X_4_^2^ are significant.

The probability values of the coefficients suggest that within the four variables studied, the interaction between X_1_ and X_4_ (CMC and FeSO_4_·7H_2_O) had a very significant effect on endoglucanase production by *Bacillus subtilis* strain Fatma/1 with a probability value of 0.012. On the other hand, the interaction between X_2_ and X_4_ (peptone and FeSO_4_·7H_2_O) had a significant effect with a probability value of 0.0213. The coefficients of interaction between X_1_ and X_2_, X_2_ and X_3_ and X_4_ are not significant model terms, thus do not contribute to the yield of endoglucanase.

Table 7 shows the fit summary results which contributed to select the highest order polynomial model where the lack of fit test is insignificant and the terms are significant, also the model summary statistics focus on the model that has a lower standard deviation and higher adjusted and predicted R-squared. The fit summary results (Table 7) showed that, the quadratic model is a highly significant and adequate model fitting the FCCCD of endoglucanase production by *Bacillus subtilis* strain Fatma/1 with a very low probability value (*P-*value < 0.0001), also lack of fit *F*-value 2.34 (the lack of fit is not significant, *P-*value = 0.1802). The summary statistics of the model showed the smallest value of standard deviation (0.41) and the largest adjusted and predicted R-squared of 0.9943 and 0.9852; respectively. The R^2^-prediction revealed that the model anticipated responses for new experiments and that the endoglucanase production by *Bacillus subtilis* strain Fatma/1 is predictable with 98.52% accuracy.

**Table 7:** Fit summary for Face-centered central composite design results for endoglucanase production by *Bacillus subtilis* strain Fatma/1 as affected by CMC, yeast extract, peptone and FeSO_4_ concentrations.

Lack of fit tests					
Source	Sum of squares	*df*	Mean square	*F-*value	*P-*value *P*rob > *F*
Linear	741.25	20	37.06	418.39	< 0.0001*
2FI	737.14	14	52.65	594.38	< 0.0001*
Quadratic	**2.07**	**10**	**0.2073**	**2.34**	**0.1802**

Sequential model sum of squares					
Source	Sum of squares	*df*	Mean square	*F-*value	*P-*value *P*rob > *F*
Linear vs Mean	110.79	4	27.70	0.93	0.4605
2FI vs Linear	4.11	6	0.68	0.02	1
Quadratic vs 2FI	**735.07**	**4**	**183.77**	**1095.54**	< 0.0001*

Model summary statistics					
Source	Standard deviation	R-squared	Adjusted R-squared	Predicted R-squared	PRESS
Linear	5.45	0.13	− 0.0092	− 0.2296	1048.22
2FI	6.23	0.1348	− 0.3206	− 2.2136	2739.54
Quadratic	**0.41**	**0.997**	**0.9943**	**0.9852**	**12.60**

*Significant values, *df* : degree of freedom, PRESS: sum of squares of prediction error, two factors interaction: 2FI.

Table 7 shows the calculations and the data of the coefficients of regression equation which were fitted to a second-order polynomial equation.

6 $$ Y = \, + 29.6--\;1.69{X_1} + 0.87{X_2}--1.55{X_3} - 0.371 \, {X_4} + 0.01{X_1}{X_2} - 0.23{X_1}{X_3} + 0.29{X_1}{X_4} - 0.03{X_2}{X_3} + 0.26{X_2}{X_4} + 0.22{X_3}{X_4}--6.31{X_1}^2--4.34{X_2}^2 + 0.67{X_3}^2--0.89{X_4}^2$$

where Y is the predicted value of endoglucanase activity by *Bacillus subtilis* strain Fatma/1, X_1_, X_2,_ X_3_ and X_4_ are the coded levels of the independent factors required for endoglucanase production by *Bacillus subtilis* strain Fatma/1.

### Three dimensional (3D) plots

The three-dimensional graph provide a method for visualizing the relationship between the interactions within test variables and the response in order to optimize the conditions for endoglucanase production by *Bacillus subtilis* strain Fatma/1. The three-dimensional plots for the significant pair-wise combinations of the four variables (X_1_ X_2_, X_1_ X_3,_ X_1_ X_4,_ X_2_ X_3,_ X_2_ X_4_ and X_3_ X_4_) were generated by plotting the response (endoglucanase activity) on Z-axis against two-independent variables while maintaining other variables at their zero levels (center points).

**Figure 6:**
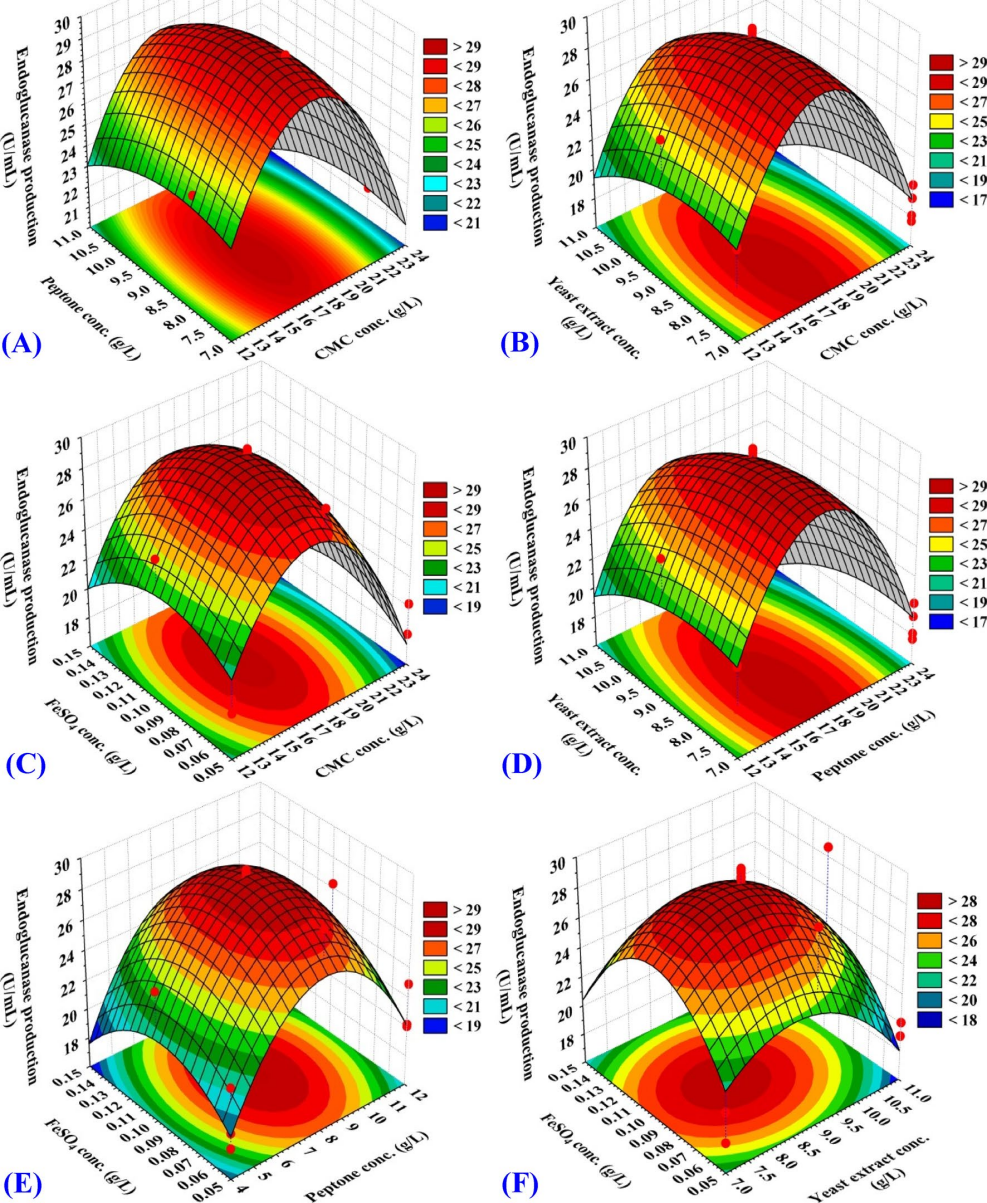
Three-dimensional surface plot for endoglucanase production by *Bacillus subtilis* strain Fatma/1 as affected by CMC, yeast extract, peptone and FeSO_4_ concentrations.

The 3D plot (Fig. 6A), illustrates the impact of CMC (X_1_) and peptone (X_2_) on endoglucanase production, while yeast extract (X_3_) and FeSO_4_.7H_2_O (X_4_) was kept at their zero levels (9 and 0.1 g/L respectively). Figure 5A shows that lower and higher levels of CMC (X_1_) support relatively lower endoglucanase activity. On the other hand, the maximum endoglucanase activity clearly situated close to the central point of both CMC (X_1_) and peptone (X_2_). By solving the Eq. (6) and analysis of Fig. 6A, the maximum predicted endoglucanase production (31.82 U/mL) was obtained at the optimum predicted levels of CMC peptone. The 3D plot (Fig. 6B), designates the impact of CMC (X_1_) and yeast extract (X_3_) on endoglucanase production while the other two variable factors were maintained at zero levels. It is evident from Fig. 6B that endoglucanase production increased when yeast extract (X_3_) was decreased to 7 g/l and at CMC (X_1_) of 18 g/L, while the two other variable factors were kept in zero levels. By solving the Eq. (6) and analysis of Fig. 6B, the maximum predicted endoglucanase production of 31.82 U/mL was obtained at the optimum predicted levels of CMC (X_1_), peptone, yeast extract, and FeSO_4_·7H_2_O, were 18, 8, 7 and 0.1 g/L respectively. The 3D plot (Fig. 6C), highlight the roles played by CMC (X_1_), and FeSO_4_·7H_2_O in endoglucanase production, when CMC (X_1_) and FeSO_4_·7H_2_O (X_4_) were kept at zero levels. The endoglucanase production was increased when the two other variable factors were kept at zero level. By solving the Eq. (6) and analysis of Fig. 6C, the maximum predicted endoglucanase production of 31.82 U/mL was obtained at the optimum predicted levels of CMC (18 g/L), peptone (8 g/L), yeast extract (7 g/L) and FeSO_4_.7H_2_O of (0.1 g/L).

The 3D plot (Fig. 6D) represent the effect of interaction between peptone and yeast extract on endoglucanase production, when peptone was at zero level and yeast extract was at its low level. Both CMC and yeast extract were kept at zero level thereafter endoglucanase production was increased. The 3D plot (Fig. 6E) investigated the impact of peptone and FeSO_4_·7H_2_O on endoglucanase yield. The endoglucanase production by *Bacillus subtilis* strain Fatma/1 was increased when peptone of 8 g/L, FeSO_4_·7H_2_O of 0.1 g/L, CMC of 18 g/L and yeast extract of 9 g/L were applied. 3D plot (Fig. 6F), denoted the impact of interactions between yeast extract and FeSO_4_·7H_2_O on the endoglucanase production by *Bacillus subtilis* strain Fatma/1, when CMC and peptone were kept at zero levels. The results indicated that the low level of yeast extract and zero level of FeSO_4_·7H_2_O enhanced endoglucanase production by *Bacillus subtilis* strain Fatma/1, while the two other factors were at their zero levels.

### Desirability function (DF)

**Figure 7:**
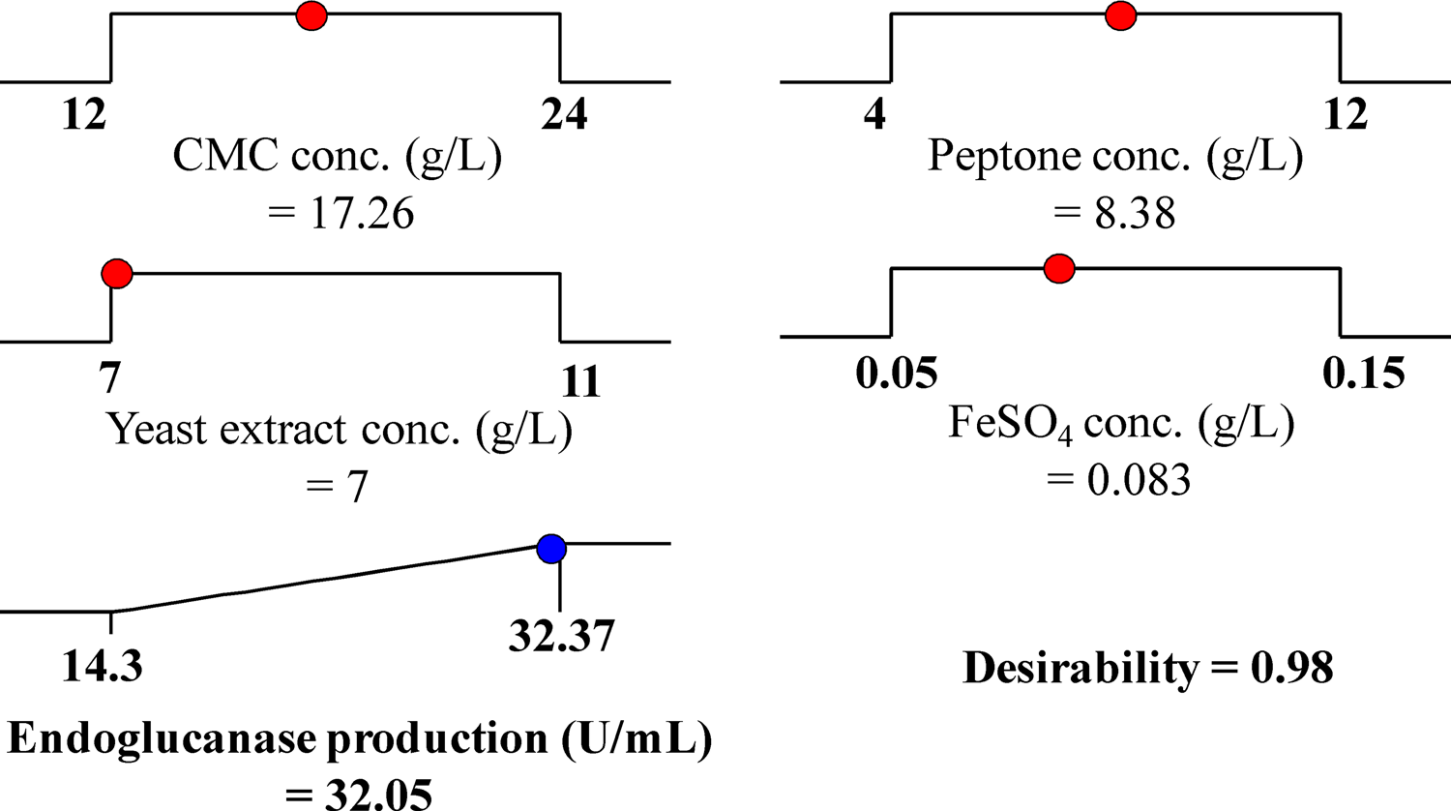
The optimization graph shows the optimal expected values and the desirability function for the maximal endoglucanase production by *Bacillus subtilis* strain Fatma/1 as affected by CMC, yeast extract, peptone and FeSO_4_ concentrations.

The purpose of the experimental design is to investigate the optimal predicted conditions required for maximizing the responses^77^. This was achieved through the desirability function (DF) option in the Design Expert Software. Values obtained ranged from zero, which is undesirable to one that is a desirable value. The numeric optimization finds the points that improve the desirability function. Figure 7 exhibits the optimization plot with the optimum predicted values and the desirability function for the highest endoglucanase production by *Bacillus subtilis* strain Fatma/1. The maximum predicted value of endoglucanase production by *Bacillus subtilis* strain Fatma/1 (32.05 U/mL) was achieved in the presence of g/L: CMC (17.26), peptone (8.38), yeast extract (7) and FeSO_4_·7H_2_O (0.083). The verification experiment showed that the experimental results and their predicted values are quietly in deep agreement suggested that the DF efficiently calculated the optimal predicted conditions for the endoglucanase production by *Bacillus subtilis* strain Fatma/1 with approximately 99.3% accuracy.

Thus, the optimum concentration of each medium component estimated by the model was verified experimentally and compared to the theoretical data. The maximum endoglucanase activity obtained was 32.42 U/mL higher than many cellulase producing *Bacillus* including *Bacillus subtilis* AS3 (0.75 U/mL), *Bacillus* sp. (1.6 U/mL), *Bacillus licheniformis* NCIM 5556 (42.99 U/mg) and *B. amyloliquefaciens* SS35 (0.528 U/mL^47,52,78,79^.

### Purification of endoglucanase enzyme

**Figure 8:**
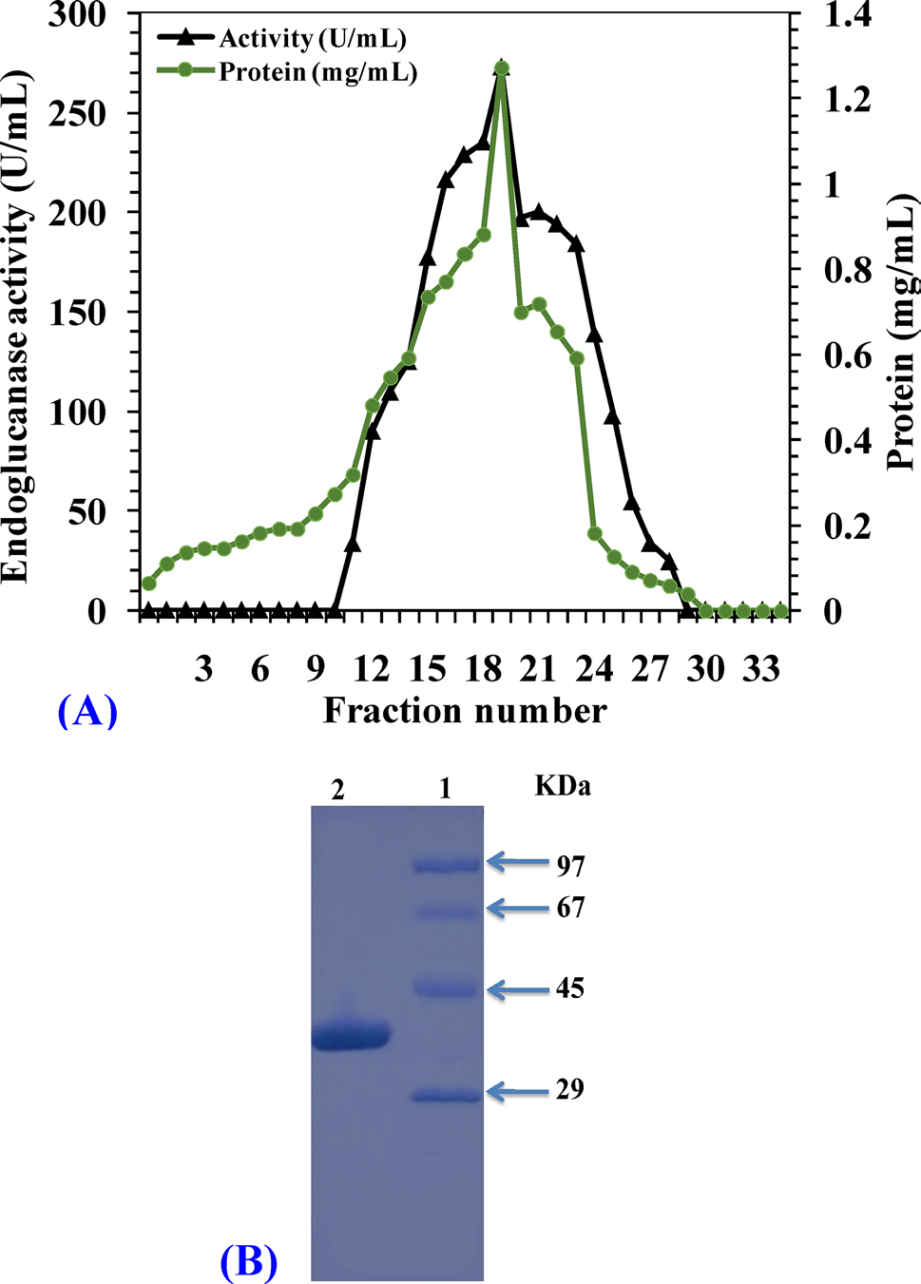
(**A**) Fractionation of endoglucanase enzyme by DEAE-Sepharose CL6B column (**B**) Molecular weight determinations by SDS-PAGE method. On the right side, marker proteins: Phosphorylase B (97 kDa), Bovine serum albumin (67 kDa), Ovalbumin (45 kDa) and Carbonic anhydrase (29 kDa). On the left side, purified protein.

Details of the endoglucanase purification are given in Table 8. The specific activity of the crude enzyme was 70.9 U/mg protein. The crude enzyme was further purified by ammonium sulfate precipitation. By determining the endoglucanase activity in ammonium sulfate fractions, the fraction with 80% saturation gave higher endoglucanase activity. Similar result was observed by El-Naggar et al*.*^80^. The enzyme gave a specific activity of 113.9 U/mg and a yield of 84.3%. Hence, there was a 1.6 purification fold. Several fractions were then obtained on subsequent purification via ion exchange chromatography; each is 3 mL in size. Figure 8A illustrates the derived protein content and the endoglucanase activity of different enzyme fractions obtained via DEAE-Sepharose column. Fractions from 12 to 27 were positive for endoglucanase activity with the highest activity shown at fraction number 20. The purification process resulted in 4.36-fold purification and a final recovery of 52.9% of the enzyme and a specific activity of 309.8 U/mg (Table 8). The activity of the purified enzyme is greater than that of endoglucanases produced by many *Bacillus* species including *Bacillus* sp. C1 (68.1 U/mg), *Bacillus* sp. JS14 (16.25 U/mg)^81,82^. Therefore, the isolated strain *Bacillus subtilis* Fatma/1 is of interest for further studies.

**Table 8:** Purification of endoglucanase activity.

Purification step	Total protein (mg)	Total activity (U)	Specific activity (U/mg protein)	Purification fold	Yield (%)
Crude extract	80	5675	70.9	1	100
Ammonium sulfate	42	4785	113.9	1.6	84.3
DEAE-Sepharose column chromatography	9.7	3005	309.8	4.36	52.9

*Specific activity(U/mg) = Total endoglucanase activity (U)/Total protein(mg).

*Purification fold = Specific activity of certain purification step /Specific activity of the crude enzyme.

*Yield (%) = (Total activity from a certain purification step / total activity of the crude enzyme) × 100.

### SDS-PAGE protein electrophoresis and determination of molecular weight of endoglucanase

SDS–PAGE separation of the enzyme preparation showed only one distinctive band. The relative mobility (R_f_) value was calculated for the distinctive single band^83^. The molecular weight of the purified enzyme was estimated to be 37 kDa compared with standard marker proteins as revealed in Fig. 8B. This is close to the molecular weight of cellulase from *B*. *amyloliquefaciens* SS35 (37 KDa)^84^, *B*. *subtilis* YJ1 (32.5 kDa)^85^ and *B*. *licheniformis* (37 kDa)^86^.

### Physicochemical properties of purified endoglucanase

The activity of endoglucanase from *Bacillus subtilis* strain Fatma/1 was characterized at different temperatures, pH levels, substrate concentrations and incubation times.

### Temperature effect on endoglucanase activity

**Figure 9:**
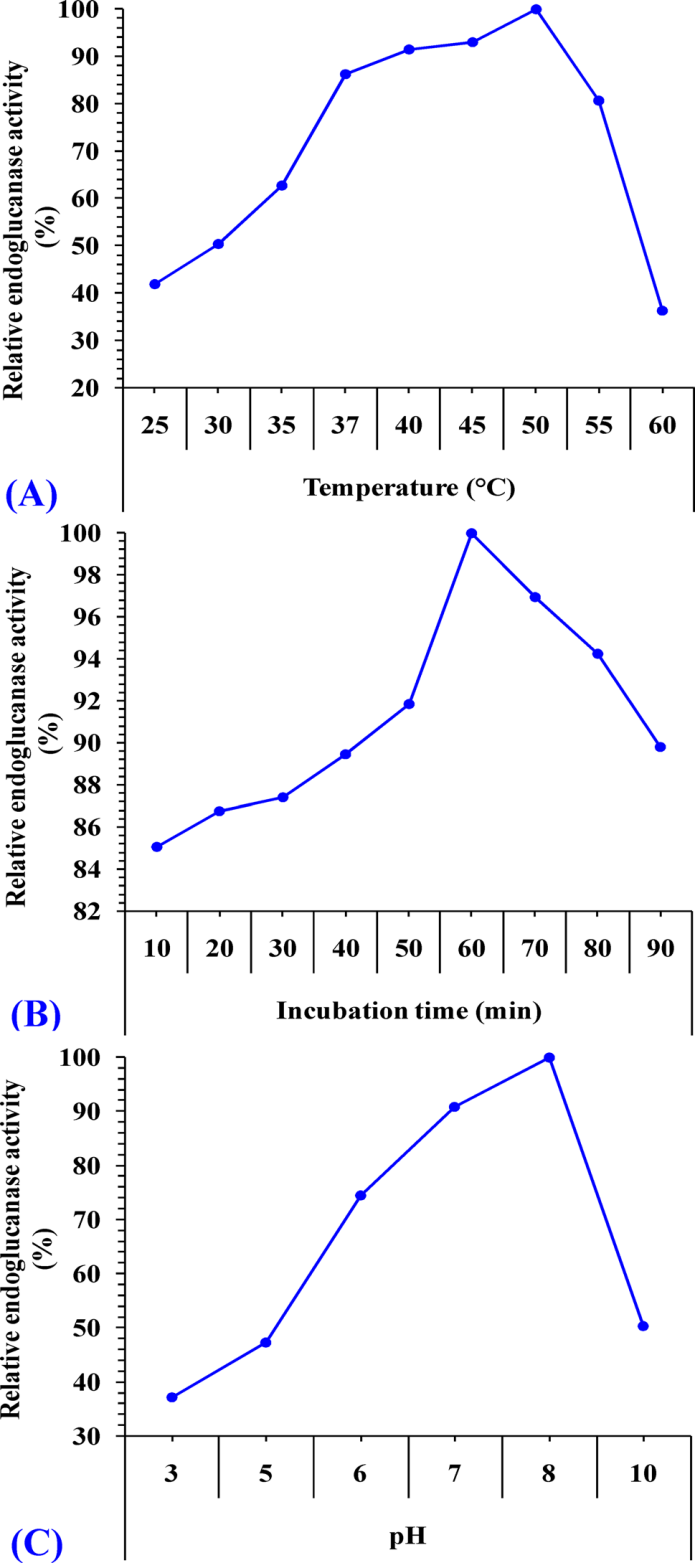
Physicochemical characterization of endoglucanase enzyme (**A**) Relative activity of endoglucanase as a function of the temperature of the reaction (**B**) Relative activity of endoglucanase as a function of the time of the reaction (**C**) Activity of endoglucanase as a function of the pH of the reaction.

The highest endoglucanase activity was recorded when incubating the reaction mixture with CMC at 50 °C. The enzyme was still active over a wide temperature range of 25–60 °C as shown in Fig. 9A. However, activity sharply reduced beyond this temperature. Similar results were obtained by Regmi et al*.*^87^ and Zubair et al*.*^88^ on their work on endoglucanase from *Bacillus Subtilis.* Dehghanikhah et al.^89^ also reported maximum endoglucanase activity at 60 °C.

### Effect of pH on endoglucanase enzyme activity

The results in Fig. 9B illustrated that endoglucanase was active over a broad pH range of 3.0 to 10.0 with highest activity at pH 8.0. The activity of endoglucanase clearly decreased at higher pH values. The enzyme retains 37.2% of its activity at pH 10.0 and 50.4% at pH 3. These results are in agreement with those obtained by Dehghanikhah et al.^89^ and Regmi et al*.*^87^ who reported maximum endoglucanase activity at pH 8 and 7.5 respectively. Irfan et al.^90^ reported an optimum pH of 7.0 while Rawat and Tewari^10^ recorded maximum enzyme activity at pH value of 4.0.

### Incubation time effect on endoglucanase activity

The activity of endoglucanase was gradually enhanced with increasing the incubation time of the reaction mixture up to 60 min. Figure 9C showed a little decrease in activity upon increasing the incubation time above 60 min. Similar finding was reported by Islam et al.^52^ who found that an incubation period of 60 min was optimum for enhancing endoglucanase activity produced by *Bacillus* sp.

### Thermal and pH stabilities of endoglucanase enzyme

**Figure 10:**
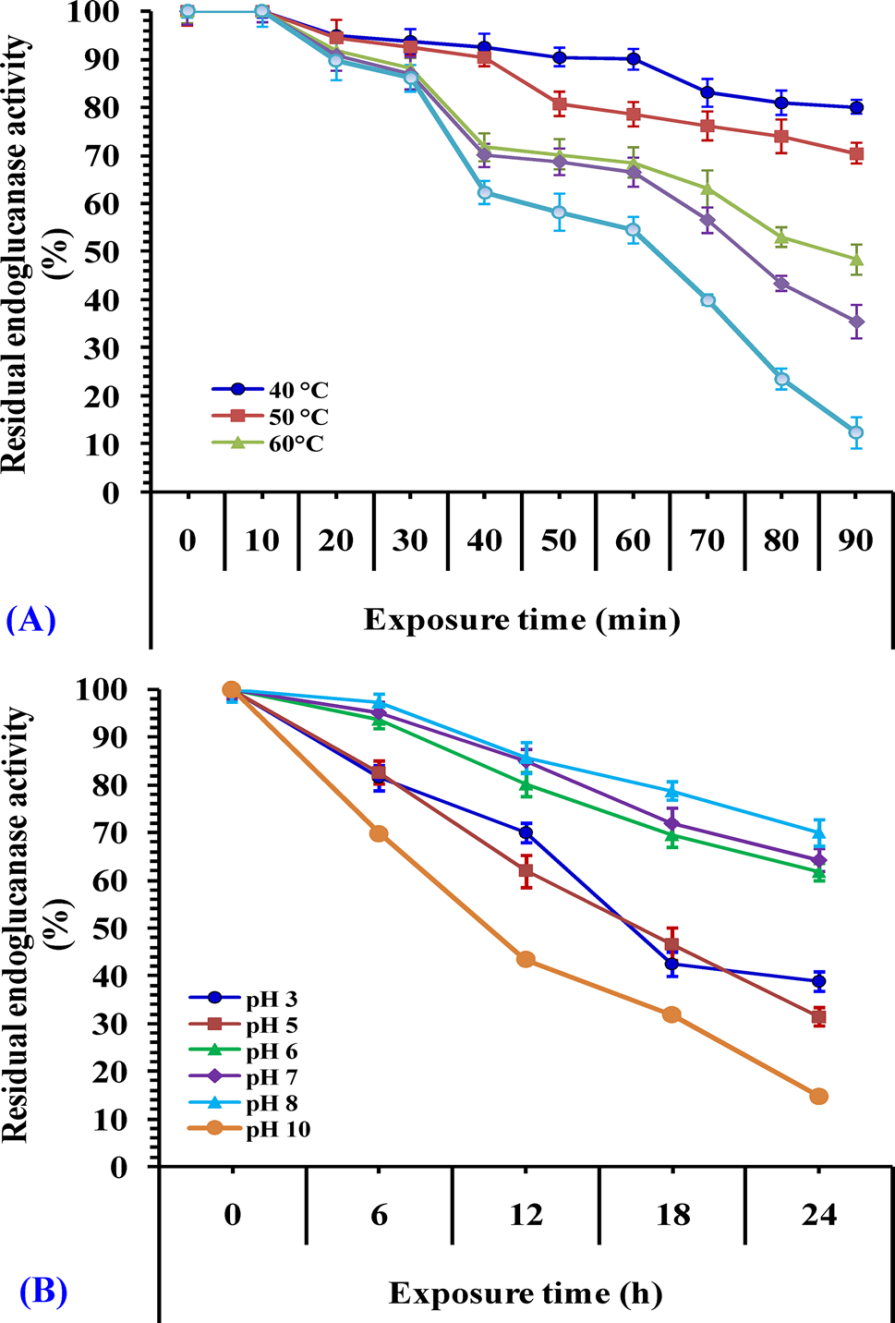
Thermal and pH stability of endoglucanase enzyme (**A**) pH stability of endoglucanase enzyme at different times of the reaction (**B**) Thermal stability of endoglucanase as a function of the time of the reaction.

The effect of temperature on enzyme activity was studied and the optimum temperature for the reaction with CMC as substrate was observed at 50 °C in 50 mM Tris–HCl (pH 8.0) with more than 35.4% of the residual activity being maintained at 70 °C. The enzyme retained 80% of its activity after being incubated at 40 °C for 90 min (Fig. 10A). Endoglucanase showed a residual activity of 70.4% after being incubated for 90 min at 50 °C. The activity of endoglucanase greatly decreased on exposure to higher temperatures with a residual activity of only 12.43% when incubated for 90 min at 80 °C.

Endoglucanase was shown to be more stable in alkaline pH (Fig. 10B). Kim et al*.*^91^ reported similar findings for endoglucanase from *Bacillus subtilis* that was much stable at pH range 6–10.

The heat inactivation half-life time (T_1/2_) of the endoglucanase was determined at the employed temperatures and heat deactivation constant (k_d_), the linear regression of the measured values was produced by fitting the data points to first order equation that is according to Eq. (4) and their values were provided in Table 9.

**Table 9:** Half-life time (T_1/2_) and heat deactivation constant (k_d_) of endoglucanase produced by *Bacillus subtilis* strain Fatma/1.

Temperature (ºC)	Half life time (min)	k_d_ (min)	*P* value	R^2^ value
40	213.95	0.0032	< 0.0001	0.9558
50	139.53	0.0050	< 0.0001	0.9708
60	82.67	0.0084	< 0.0001	0.9691
70	68.05	0.0102	< 0.0001	0.9697
80	49.42	0.0140	< 0.0001	0.9699

The half-life time (T_1/2_) of endoglucanase was reported to be 139.53 min at 50 °C and 82.67 min at 60 °C. However, the devastation of endoglucanase enzyme was observed at 80 °C with short half-life time (49.42 min). The endoglucanase enzyme activity or residual activity after exposure to different temperatures for varied times was compared to the initial activity of the enzyme before exposure which was considered as the control or 100%. Endoglucanase was active over a wide temperature and pH range with an optimum activity at 50 °C and pH value 8. Enzyme deactivation at different temperatures and pH values is considered a major restriction in the process of selecting enzymes for industrial and biotechnological purposes.

Endoglucanase produced by *Bacillus subtilis* strain Fatma/1 has a good activity over a wide range of temperature and pH values and its significant thermal and pH stabilities, makes it suitable for biotechnological and industrial purposes.

### Tube method and microscopic observation of biofilms

**Figure 11:**
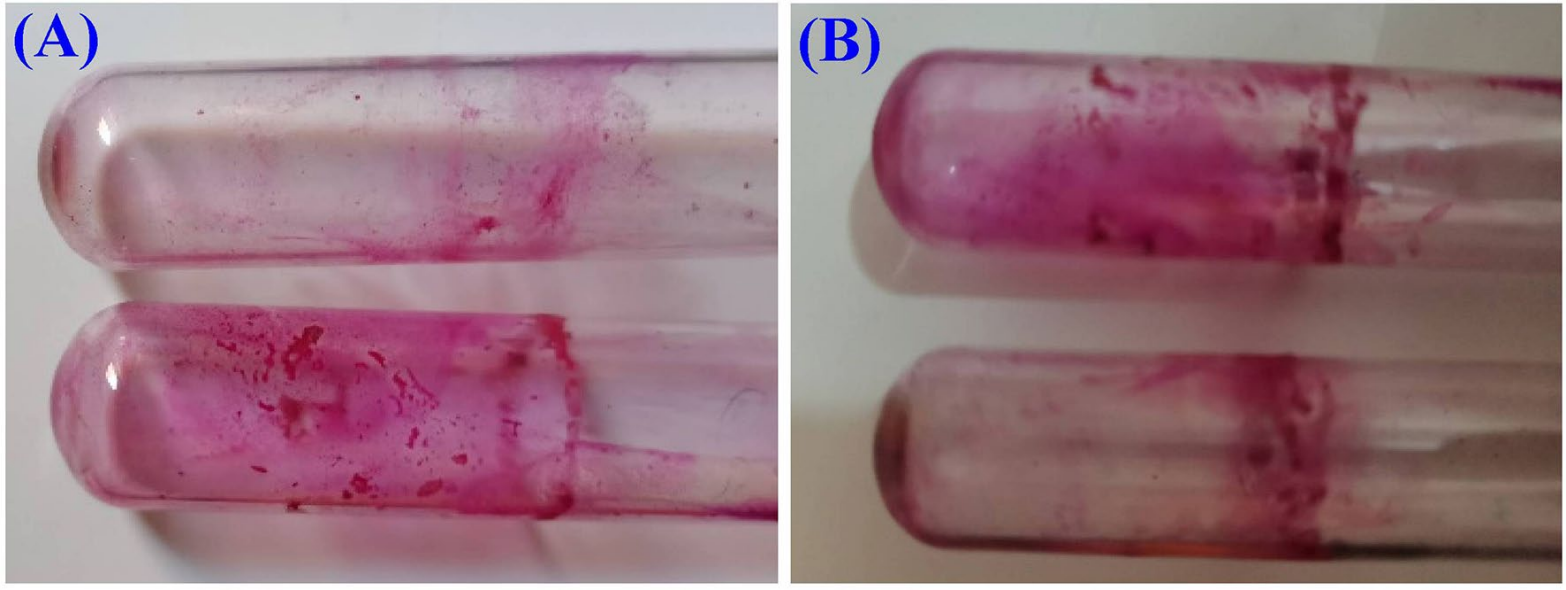
Evaluation of biofilm degradation ability of endoglucanase based on color intensity of the adherent safranin ring (**A**) *P. aeruginosa* biofilm treated and control (**B**) *S. aureus* biofilm treated and control.

The Biofilm degradation ability of endoglucanase was evaluated by tube method and endoglucanase exhibited high efficiency in removing biofilm formed by *P. aeruginosa* and moderate ability in removing *S. aureus* biofilms (Fig. 11).

**Figure 12:**
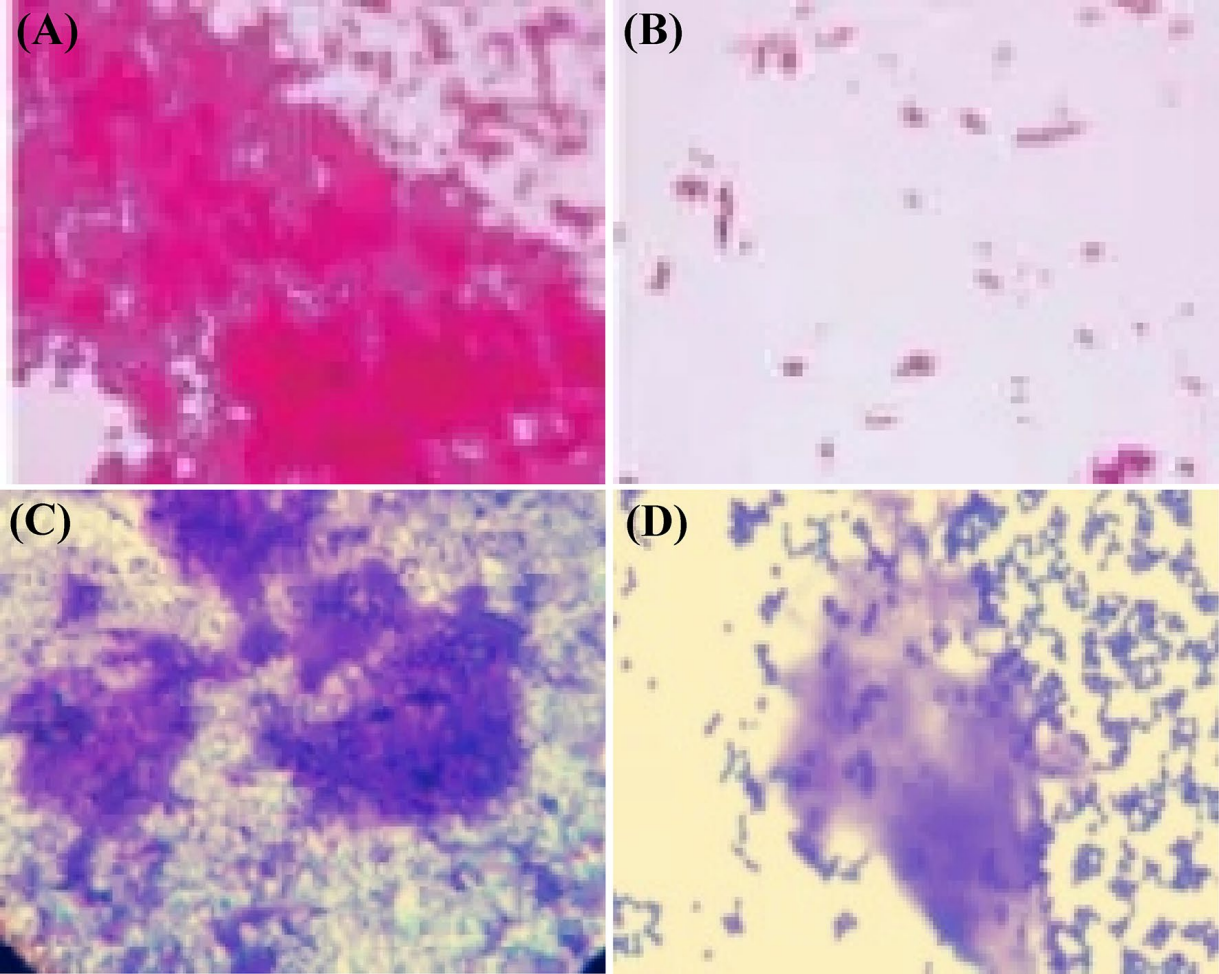
Microscopic examination (40×) of anti-biofilm activity of endoglucanase enzyme on (**A**) *P. aeruginosa* biofilm untreated (**B**) *P. aeruginosa* biofilm treated (**C**) *S. aureus* biofilm untreated (**D**) *S. aureus* biofilm treated.

Stained glass pieces were placed on slides and examined for biofilm growth or dispersion by light microscopy. Slides of *P. aeruginosa* with enzymatic treatments revealed dispersed planktonic cells while treated slides of *S. aureus* biofilms revealed partial dispersion of the biofilm matrix as illustrated in Fig. 12.

### The effect of endoglucanase on different species biofilms

**Figure 13:**
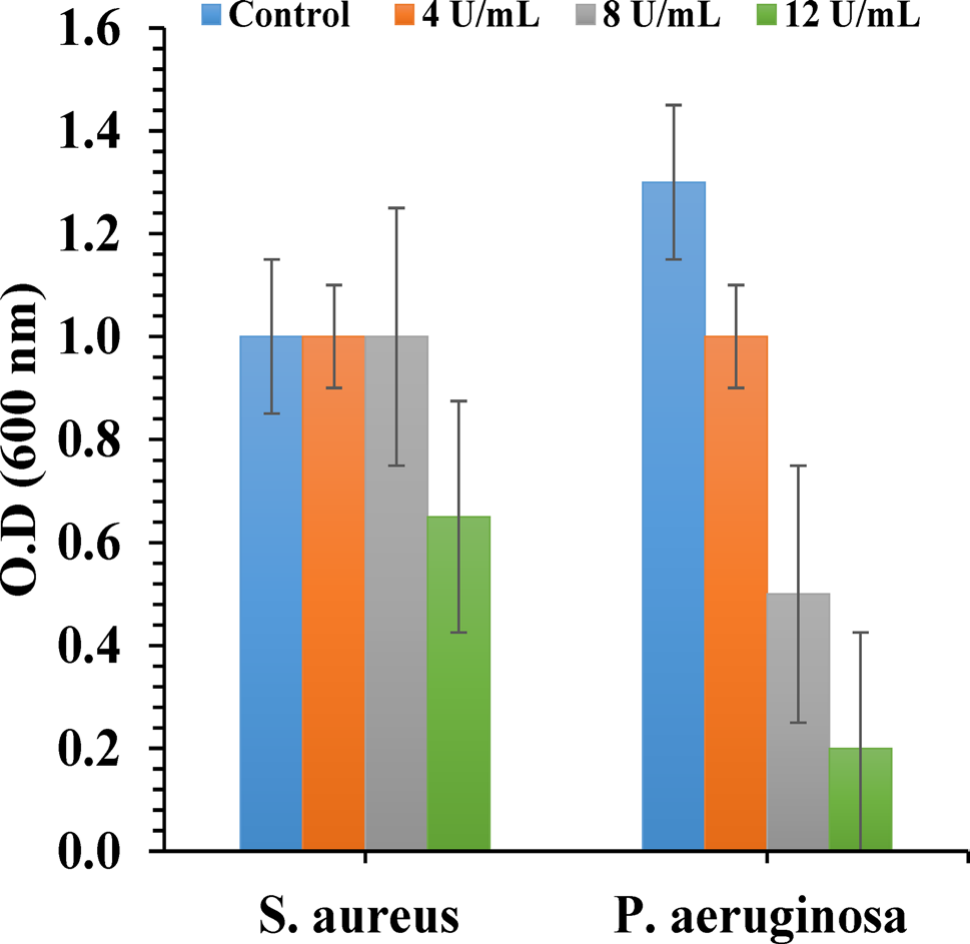
Biofilm dispersion of *S. aureus* and *P. aeruginosa* after 24 h of enzymatic treatments by crystal violet assay.

The ability of endoglucanase to disrupt different species biofilms grown in BHI was determined. Biofilms of *P. aeruginosa* treated with different concentrations of endoglucanase were greatly reduced while *S. aureus* biofilms was shown to be more resistant to first two concentrations of endoglucanase (4–8 U/mL) with an obvious reduction in biofilm only at 12 U/mL (Fig. 13).

It has been proposed that removing cells from the protective shield of a biofilm will render them more susceptible to antimicrobial agents and the host immune response^92^**.** The effective degradation of *P. aeruginosa* biofilms by cellulase also have been reported by many studies^25,93^. As microorganisms normally generate and use bio enzymes for the breakdown and dispersal of biofilms, their use is an intuitive mechanism^92^**.**

### Bactericidal effect of endoglucanase on planktonic cells

Endoglucanase exhibited no antibacterial effect on strains used in this study. No clear zones appeared around any disc of any enzyme concentration. Similar finding was reported by Trizna et al*.*^94^ on his studies on *P. aeruginosa*.

### Estimation of total carbohydrate content of biofilms

The total carbohydrate content of bacterial biofilms was assayed before and after enzymatic treatments with endoglucanase. Results in Table 10 revealed a marked reduction in the carbohydrate content of the biofilm of *Pseudomonas aeruginosa* from 63.4 to 7.9 μg after enzymatic treatment. This indicates the efficacy of endoglucanase enzyme in eliminating the biofilm.

**Table 10:** Comparison of carbohydrate content of biofilm of different strains before and after enzyme treatment.

Organism	With enzyme	Control-without enzyme
	Carbohydrate (μg)	Carbohydrate (μg)
*S*. *aureus*	56.24 ± 0.08	106.7 ± 0.43
*P*. *aeruginosa*	7.9 ± 0.06	63.4 ± 0.5

Endoglucanase at concentration of 12 U/mL effectively removed 84.61% of biofilm matrix of *P. aeruginosa* while only removed about 30% of *S. aureus*`s biofilm*.* The effectiveness of biofilm degrading enzymes relies mainly on the composition of the biofilm matrix^95^. Different enzymes or a mixture of enzymes should be used with different biofilms in respect to their main constituents^96^.
